# Tim-3 mediates T cell trogocytosis to limit antitumor immunity

**DOI:** 10.1172/JCI152864

**Published:** 2022-05-02

**Authors:** Ornella Pagliano, Robert M. Morrison, Joe-Marc Chauvin, Hridesh Banerjee, Diwakar Davar, Quanquan Ding, Tokiyoshi Tanegashima, Wentao Gao, Saranya R. Chakka, Richelle DeBlasio, Ava Lowin, Kevin Kara, Mignane Ka, Bochra Zidi, Rada Amin, Itay Raphael, Shuowen Zhang, Simon C. Watkins, Cindy Sander, John M. Kirkwood, Marcus Bosenberg, Ana C. Anderson, Vijay K. Kuchroo, Lawrence P. Kane, Alan J. Korman, Arvind Rajpal, Sean M. West, Minhua Han, Christine Bee, Xiaodi Deng, Xiao Min Schebye, Pavel Strop, Hassane M. Zarour

**Affiliations:** 1 Department of Medicine and UPMC Hillman Cancer Center,; 2 Department of Computational and Systems Biology, School of Medicine,; 3 Department of Immunology, and; 4 Department of Cell Biology, University of Pittsburgh, Pittsburgh, Pennsylvania, USA.; 5 Departments of Dermatology, Pathology, and Immunobiology, Yale University School of Medicine, New Haven, Connecticut, USA.; 6 Evergrande Center for Immunologic Diseases and Ann Romney Center for Neurologic Diseases, Harvard Medical School and Brigham and Women’s Hospital, Boston, Massachusetts, USA.; 7 Biologics Discovery California, Bristol Myers Squibb, Redwood City, California, USA.

**Keywords:** Immunology, Oncology, Cancer immunotherapy, Melanoma, T cells

## Abstract

T cell immunoglobulin mucin domain-containing protein 3 (Tim-3) negatively regulates innate and adaptive immunity in cancer. To identify the mechanisms of Tim-3 in cancer immunity, we evaluated the effects of Tim-3 blockade in human and mouse melanoma. Here, we show that human programmed cell death 1–positive (PD-1^+^) Tim-3^+^CD8^+^ tumor-infiltrating lymphocytes (TILs) upregulate phosphatidylserine (PS), a receptor for Tim-3, and acquire cell surface myeloid markers from antigen-presenting cells (APCs) through transfer of membrane fragments called trogocytosis. Tim-3 blockade acted on Tim-3^+^ APCs in a PS-dependent fashion to disrupt the trogocytosis of activated tumor antigen–specific CD8^+^ T cells and PD-1^+^Tim-3^+^ CD8^+^ TILs isolated from patients with melanoma. Tim-3 and PD-1 blockades cooperated to disrupt trogocytosis of CD8^+^ TILs in 2 melanoma mouse models, decreasing tumor burden and prolonging survival. Deleting Tim-3 in dendritic cells but not in CD8^+^ T cells impeded the trogocytosis of CD8^+^ TILs in vivo. Trogocytosed CD8^+^ T cells presented tumor peptide–major histocompatibility complexes and became the target of fratricide T cell killing, which was reversed by Tim-3 blockade. Our findings have uncovered a mechanism Tim-3 uses to limit antitumor immunity.

## Graphical abstract

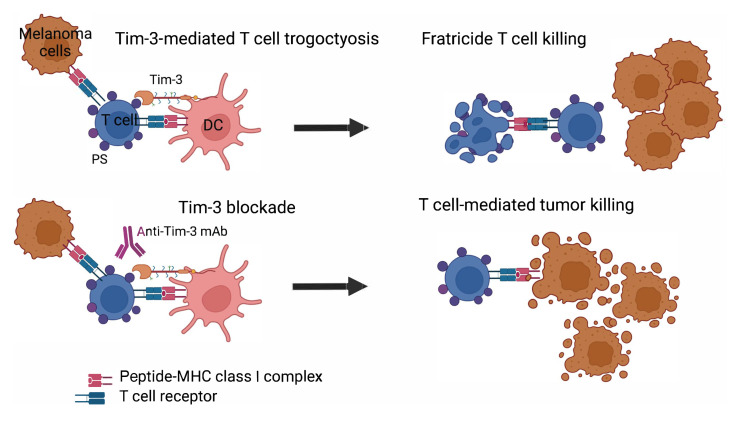

## Introduction

During chronic viral infection and cancer, tumor antigen–specific (TA-specific) CD8^+^ T cells become dysfunctional and upregulate multiple inhibitory receptors, including programmed cell death 1/programmed death ligand 1 (PD-1/PD-L1) and cytotoxic T lymphocyte–associated antigen 4 (CTLA-4; refs. 1, 2). Immune checkpoint blockade targeting PD-1/PD-L1 and CTLA-4, alone or in combination, confers significant clinical benefits in multiple tumor types, including melanoma (3–5). However, the majority of patients with cancer do not respond to dual PD-1 and CTLA-4 blockade, a treatment that often causes serious adverse events. Therefore, novel treatment strategies are needed, and targeting novel nonredundant inhibitory receptor pathways contributing to tumor-induced T cell dysfunction in the tumor microenvironment (TME) may prove efficacious and nontoxic (6).

T cell immunoglobulin mucin domain-containing protein 3 (Tim-3) is a negative regulator of innate and adaptive immune responses and plays a role in cancer immunity (7–9). PD-1^+^Tim-3^–^ progenitor/stem cell–like exhausted T cells differentiate into PD-1^+^Tim-3^+^ CD8^+^ T cells and play a critical role in chronic viral infections and antitumor immunity (10, 11). PD-1^+^Tim-3^+^ CD8^+^ T cells produce fewer cytokines than PD-1^+^Tim-3^–^ CD8^+^ T cells upon stimulation with cognate antigen but paradoxically exhibit superior lytic capacity and antitumor reactivity in vivo (11, 12). PD-1^+^Tim-3^+^ CD8^+^ tumor-infiltrating lymphocytes (TILs) are enriched in tumor-reactive CD8^+^ T cells (13). Dual PD-1 and Tim-3 blockade augments the expansion and function of TA-specific CD8^+^ T cells and induces tumor regression in multiple mouse tumor models (8). This combinatorial immune checkpoint blockade is now being evaluated in clinical trials for many cancers (14). Tim-3 is upregulated by multiple cell subsets in the TME, including CD8^+^ and CD4^+^ T cells, CD4^+^ regulatory T cells, and antigen-presenting cells (APCs) (15). Multiple Tim-3 ligands have been identified, including galectin-9, phosphatidylserine (PS), carcinoembryonic antigen-related cell adhesion molecule 1, and high mobility group box protein 1 (HMGB1; refs. 7, 16–20). Human CD8^+^ T cells can reversibly upregulate PS upon antigen recognition (21). T cells upregulate cell surface expression of PS upon antigen encounter, and it has been hypothesized that this serves as a protective mechanism for the cytotoxic T cell to prevent self-cytotoxicity upon degranulation (22). Multiple antigen-independent and antigen-dependent activation stimuli promote PS expression by viable CD8^+^ T cells, which exhibit antitumor cytotoxic activity in vitro and in vivo (23).

The mechanisms that Tim-3 uses to modulate cancer immunity are not fully understood. Although preclinical studies initially suggested that Tim-3 blockade acts on Tim-3^+^CD8^+^ TILs in combination with PD-1 blockade (8), there is growing evidence that Tim-3 acts in dendritic cells (DCs) to regulate antitumor immunity. In mouse mammary carcinoma treated with paclitaxel, Tim-3 appears to limit activation of the cGMP-AMP synthase/stimulator of interferon genes (cGAS/STING) pathway in intratumor DCs by suppressing extracellular DNA uptake. In this experimental model, Tim-3 blockade enhances antitumor CD8^+^ T cell–dependent response in a galectin-9–dependent fashion (24, 25). In tumor-bearing mice, loss of Tim-3 prevents DCs from expressing a regulatory program and facilitates the maintenance of CD8^+^ effector and stem-like T cells (26). Tim-3 deletion in DCs leads to increased accumulation of reactive oxygen species, resulting in NLRP3 inflammasome activation, while inhibition of inflammasome activation, or downstream effector cytokines interleukin-1β (IL-1β) and IL-18, abrogates the protective antitumor immunity observed with Tim-3 deletion in DCs. Our findings add to these preclinical studies to support that Tim-3 acts in DCs to augment antitumor immunity in mice and in humans. Our results show that Tim-3 on APCs cooperates with PS on TA-specific CD8^+^ T cells and CD8^+^ TILs to limit antitumor immunity through T cell trogocytosis and fratricide killing.

## Results

### PD-1^+^Tim-3^+^ CD8^+^ TILs undergo trogocytosis.

**Figure 1:**
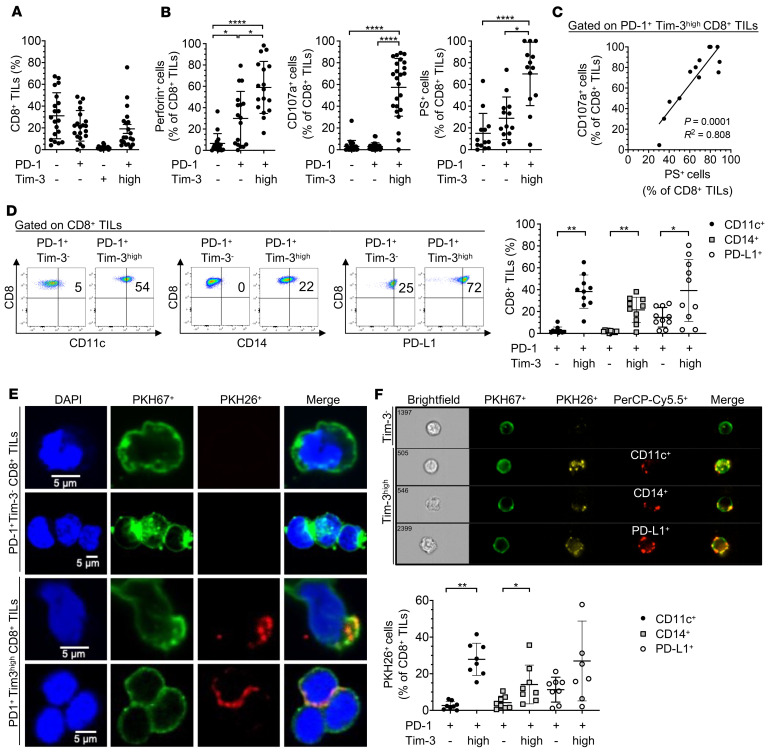
PD-1^+^Tim-3^hi^ CD8^+^ TILs express myeloid markers acquired from APCs via trogocytosis in metastatic melanoma. (**A**) Summary data (*n* = 20) showing expression of PD-1 and Tim-3 on CD8^+^ TILs in metastatic melanoma (MM). (**B**) Summary data showing frequencies of perforin^+^ (*n* = 16), CD107a^+^ (*n* = 24), and PS^+^ (*n* = 13) CD8^+^ TILs in MM according to PD-1 and Tim-3 expression. (**C**) Pooled data (*n* = 11) showing the correlation between CD107a and PS expression in PD-1^+^Tim-3^hi^ CD8^+^ TILs in MM. (**D**) Representative dot plots (left) and summary data (right, *n* = 10) showing frequencies of CD11c^+^, CD14^+^, and PD-L1^+^ CD8^+^ TILs according to PD-1 and Tim-3 expression. (**E**) Confocal micrographs of PKH67^+^ PD-1^+^Tim-3^–^ and PD-1^+^Tim-3^hi^ CD8^+^ TILs coincubated for 30 minutes with autologous PKH26^+^CD45^+^CD3^–^ cells. (**F**) Representative ImageStream analysis (upper) and summary data of cell frequencies with flow cytometry (lower, *n* = 8) showing cell surface coexpression of PKH26 and CD11c, CD14, or PD-L1 on PKH67^+^ PD-1^+^Tim-3^–^ and PD-1^+^Tim-3^hi^ CD8^+^ TILs after 30 minutes’ incubation with autologous PKH26^+^CD45^+^CD3^–^ cells. *P* values were obtained by 1-way ANOVA followed by Holm-Šídák multiple comparisons test and Friedman’s test followed by Dunn’s multiple-comparison test (**B**), simple linear regression test (**C**), and paired *t* test and Wilcoxon’s matched pairs signed-rank test (**D** and **F**). *P < 0.05; **P < 0.01; ****P < 0.0001. Data indicate mean ± SD.

**Figure 2:**
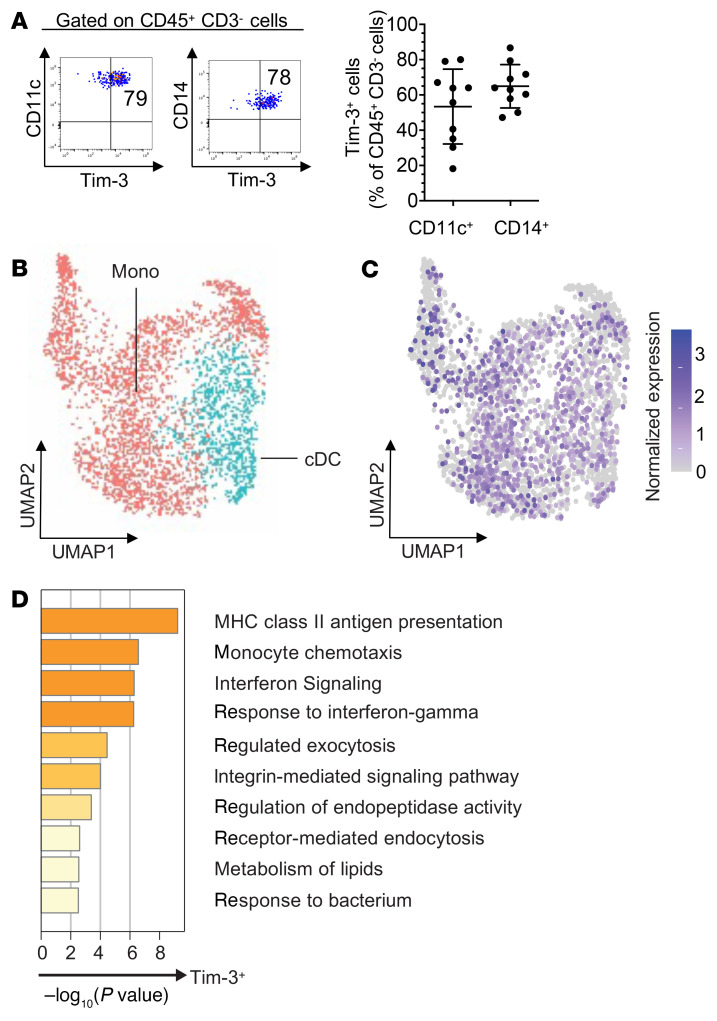
Transcriptomic features of Tim-3^+^ tumor-infiltrating myeloid cells using single-cell RNA sequencing. (**A**) Summary data showing frequencies of Tim-3^+^CD11c^+^ and Tim-3^+^CD14^+^ CD45^+^CD3^–^ cells in MM (*n* = 10). (**B**) Single-cell RNA sequencing (scRNA-Seq) of CD45^+^ cells sorted from 18 PD-1–naive MM cases. Uniform manifold approximation and projection of 3872 myeloid cells shown in dots. Monocyte (Mono) and conventional dendritic cell (cDC) clusters were identified. (**C**) Heatmap of normalized *HAVCR2* (Tim-3) expression. (**D**) Summary of pathway enrichment analysis using Metascape. Myeloid cells were separated into *HAVCR2*^+^ and *HAVCR2*^–^ groups.

We first evaluated the phenotype and function of PD-1^+^Tim-3^hi^ CD8^+^ T cells isolated from metastatic melanoma (MM), which produced fewer cytokines (TNF and IL-2) upon stimulation with phorbol myristate acetate (PMA)/ionomycin ex vivo (Supplemental Figure 1, A and B; supplemental material available online with this article; https://doi.org/10.1172/JCI152864DS1) and spontaneously exhibited higher degranulating capability (CD107a, perforin) and upregulated PS (annexin V staining) as compared with PD-1^+^Tim-3^–^ CD8^+^ TILs (Figure 1, A and B, and Supplemental Figure 1C). CD107a expression positively correlated with PS expression in PD-1^+^Tim-3^hi^ CD8^+^ TILs (Figure 1C). We evaluated PS expression together with active caspase-3 by CD8^+^ TILs and found that the majority of PS^+^PD-1^+^Tim-3^+^ CD8^+^ TILs in human MM were not apoptotic (i.e., they did not express active caspase-3), in line with previous publications of melanoma and other solid tumors (refs. 21, 23 and Supplemental Figure 1F). We also observed that a subset of PS^+^CD8^+^ TILs coexpressed PS and active caspase-3, corresponding to T cells undergoing cell death. PD-1^+^Tim-3^hi^ CD8^+^ TILs upregulated multiple myeloid markers, including CD14, CD11c, CD209, and PD-L1, which can be acquired by activated TA-specific CD8^+^ T cells in vitro from APCs upon rapid membrane transfer called trogocytosis (refs. 27–30; Figure 1, D and F; and Supplemental Figure 1D). To determine whether trogocytosis promotes the expression of myeloid markers by PD-1^+^Tim-3^hi^ CD8^+^ TILs ex vivo, we coincubated PKH67-labeled PD-1^+^Tim-3^hi^ or PD-1^+^Tim-3^–^ CD8^+^ TILs with PKH26-labeled autologous non-CD3^+^ T cells for 30 minutes before confocal microscopy, ImageStream analysis, and flow cytometry. PD-1^+^Tim-3^hi^ but not PD-1^+^Tim-3^–^ CD8^+^ TILs acquired membrane fragments from autologous non-CD3^+^ cells (Figure 1, E and F). ImageStream analyses also showed that PD-1^+^Tim-3^hi^ CD8^+^ TILs coincubated with autologous CD45^+^CD3^–^ cells acquired cell surface myeloid markers, including CD11c, CD14, CD209, and PD-L1 (Figure 1F). The vast majority of CD14^+^ and CD11c^+^ cells present at low frequency in MM upregulated Tim-3 (Figure 2A and Supplemental Figure 1E). Unsupervised clustering analysis of single-cell RNA sequencing (scRNA-Seq) of CD45^+^ cells sorted from 18 PD-1–naive MMs identified multiple myeloid cell clusters that merged into 2 main clusters: conventional DCs (cDCs) and monocytes (Mono) (Figure 2B; Supplemental Figure 2, A–C; and Supplemental Tables 1 and 2). The vast majority of both cDC and Mono clusters expressed hepatitis A virus cellular receptor 2 (*HAVCR2*) (Figure 2C and Supplemental Table 3). *HAVCR2^+^* myeloid cells upregulated transcripts involved in MHC class II antigen presentation, monocyte chemotaxis, IFN signaling, exocytosis, and receptor-mediated endocytosis, suggesting they are recruited in the TME to contribute to antigen uptake and presentation (Figure 2D). Collectively, our findings show that PD-1^+^Tim-3^+^ CD8^+^ TILs in MM exhibit high cytolytic functions, upregulate PS expression, and acquire membrane fragments from myeloid cells through trogocytosis. Tim-3^+^ tumor-infiltrating myeloid cells exhibit a transcriptional profile supporting higher antigen uptake and presentation capacities.

### Tim-3 acts on APCs in a PS-mediated fashion to induce T cell trogocytosis.

**Figure 3:**
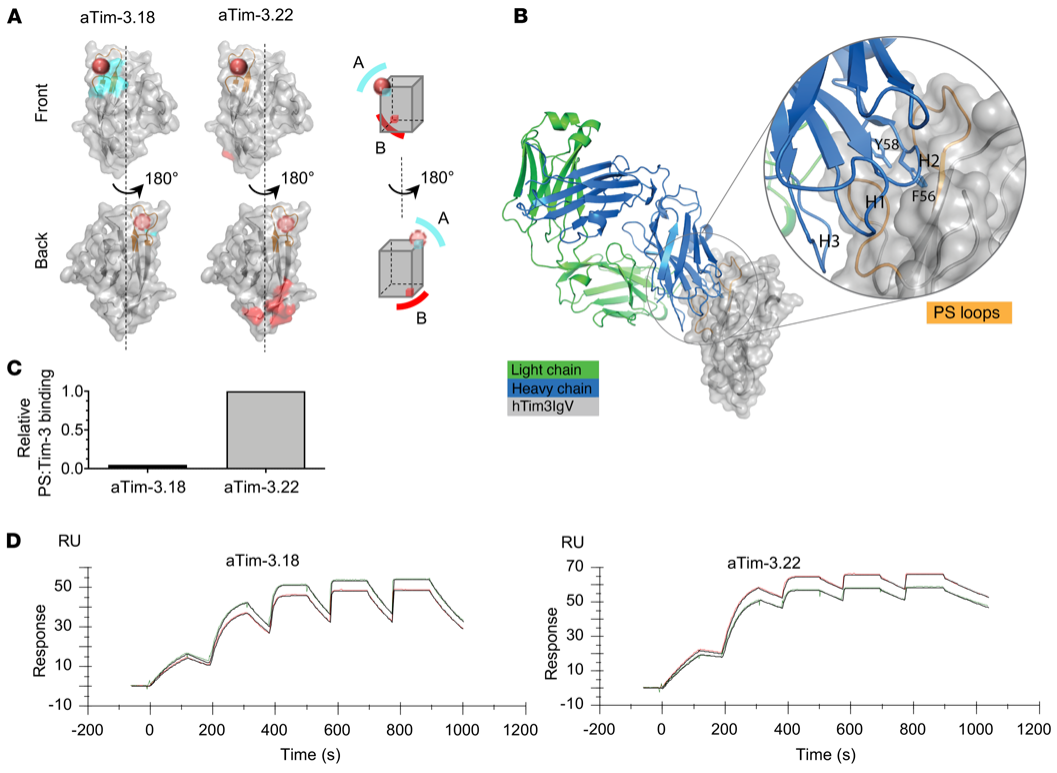
Two anti–Tim-3 antibodies bind with high affinity to Tim-3 but target 2 nonoverlapping epitopes. (**A**) Yeast display epitope mapping of aTim-3 mAbs to human Tim-3 (hTim-3): aTim-3.18 mAb binds to hTim-3 (**A**, shown in cyan) on the PS binding site (orange sphere), while aTim-3.22 binds the opposite face of hTim-3 (**B**, red) away from the PS binding site. (**B**) X-ray crystal structure of aTim-3.18:hTim-3 complex reveals heavy chain CDR2 (blue) binds the PS binding loops (orange), with residues F56 and Y58 inserted into the PS binding pocket. CDR2, complementarity-determining region 2; h, human; IgV, Ig variable. (**C**) Cell-free PS blocking assay showing relative binding of PS liposomes to hTim-3 saturated with aTim-3.18 or aTim-3.22. (**D**) Kinetic affinity measurements of aTim-3.18 and aTim-3.22 mAbs. Surface plasmon resonance sensorgrams of hTim-3 binding in increasing concentrations to aTim-3.18 (left) or aTim-3.22 (right) mAbs (data in color, fit in black). Each mAb was captured in duplicate on different flow cells.

We next wanted to assess the role of Tim-3 in regulating trogocytosis of TA-specific CD8^+^ T cells and CD8^+^ TILs in melanoma, which upregulate PS (21). To this end, we engineered 2 anti- (a) Tim-3 monoclonal antibodies (mAbs) that bind (aTim-3.18) or do not bind (aTim-3.22) the PS binding site (Figure 3, A–C, and Supplemental Table 4). The structure of aTim-3.18 fragment antigen-binding (Fab) in complex with hTim-3 IgV domain was solved at 1.5 Å using x-ray crystallography. The structure of the hTim-3 IgV domain fits well with the published hTim-3 (Protein Data Bank [PDB]: 5F71) and mouse Tim-3 (PDB: 3KAA) structures that were solved in complex with PS. Through these structural alignments, we inferred the PS binding pocket in hTim-3 (Figure 3A). The location of the PS binding pocket is conserved among the TIM members in humans and mice (31). We observed that the aTim-3.18 Fab epitope overlapped with the PS binding pocket. Specifically, the heavy chain CDR2 of aTim-3.18 occupied the PS binding pocket (Figure 3B). Additional contacts with the PS binding loops were made by heavy chains CDR1 and CDR3. Although both aTim-3.18 and aTim-3.22 mAbs bound with high affinity to Tim-3 as determined by surface plasmon resonance (Figure 3D and Supplemental Table 5), aTim-3.18 but not aTim-3.22, which binds the opposite face of Tim-3, blocked PS binding to Tim-3 (Figure 3C).

**Figure 4:**
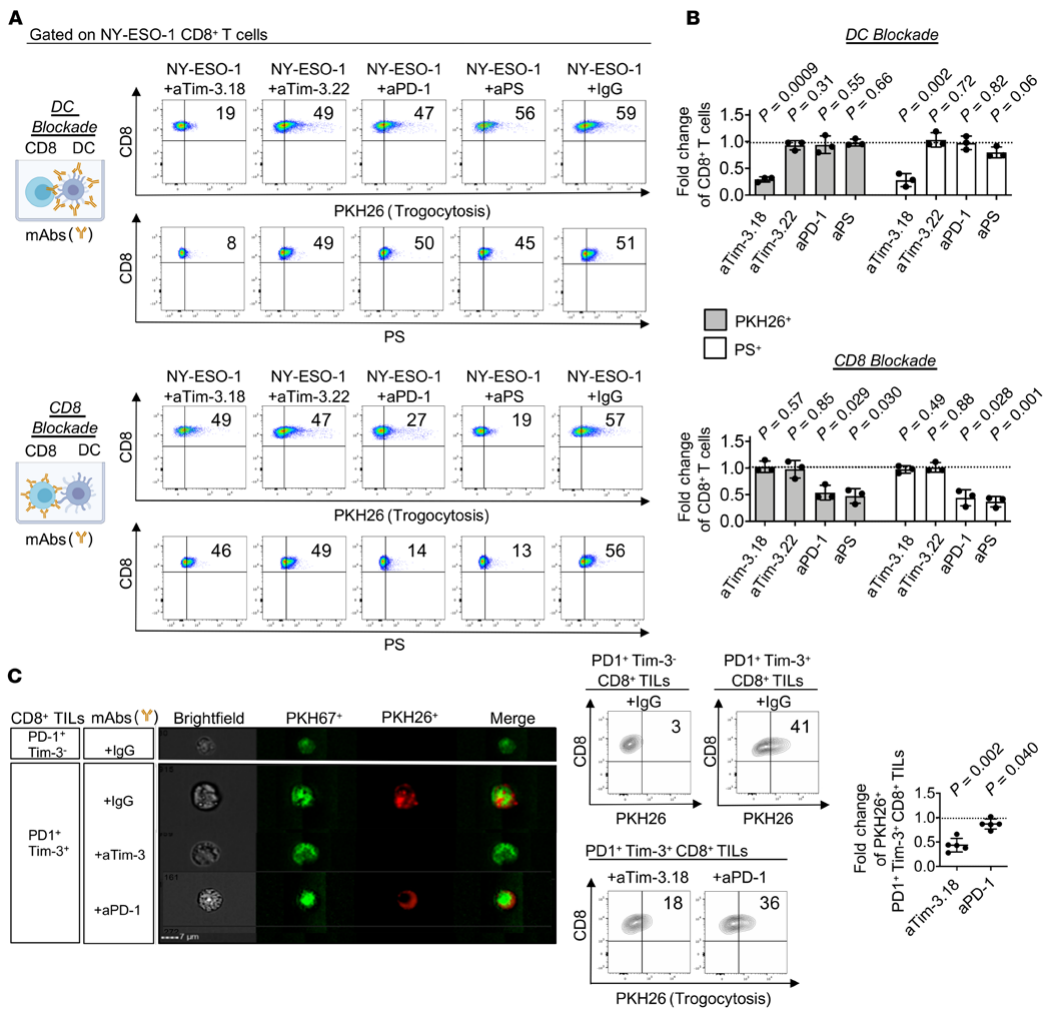
Tim-3 blockade acts in APCs in a PS-mediated fashion and cooperates with PD-1 blockade to decrease trogocytosis of TA-specific CD8^+^ T cells. (**A** and **B**) Representative flow cytometry dot plots (**A**) and summary data (**B**) evaluating PKH26 and PS expression by CD8^+^ T cells. PKH26^+^HLA-A2^+^ DCs were incubated with indicated antibodies, then added to wells with NY-ESO-1 157-165–specific clone 95/3 and cognate peptide for 5 days before flow cytometry (**A**, upper); CD8^+^ T cell clone 95/3 was incubated with indicated antibodies, then added to wells with PKH26^+^HLA-A2^+^ DCs pulsed with cognate peptide for 5 days before flow cytometry (**A**, lower). Summary data showing fold changes of PKH26^+^ and PS^+^ CD8^+^ T cells upon DC blockade (**B**, upper) or CD8^+^ T cell blockade (**B**, lower) as compared with IgG mAbs. Data shown are representative of 3 independent experiments. (**C**) Representative ImageStream images (left) and flow cytometry analysis of PKH26^+^ CD8^+^ T cells (middle and right panels). PKH67^+^ PD-1^+^Tim-3^–^ and PD-1^+^Tim-3^+^ CD8^+^ TILs were incubated for 30 minutes with PKH26^+^CD45^+^CD3^–^ cells isolated from MM in the presence of indicated antibodies before analysis. Representative summary data showing fold change of PKH26^+^ PD-1^+^Tim-3^+^ CD8^+^ TILs in wells with aTim-3.18 or aPD-1 as compared with IgG control (*n* = 5). *P* values shown (**B** and **C**) were obtained from paired *t* tests in Supplemental Figure 3, A and B. Data indicate mean ± SD.

To perform DC or CD8^+^ T cell blockade, respectively, we added PHK26-labeled DCs prepared from HLA-A2^+^ healthy donors (HDs) or HLA-A2/NY-ESO-1 157-165–specific CD8^+^ T cell clone 95/3 (32) to wells with aTim-3.18, aTim-3.22, aPD-1, aPS, or IgG control mAbs. Cells were washed before coincubation with clone 95/3 or PHK26^+^HLA-A2^+^ DCs, respectively, as well as cognate antigen for 5 days before flow cytometry (Figure 4, A and B, and Supplemental Figure 3A). aTim-3.18 but no aTim-3.22 or aPS mAbs decreased the frequencies of trogocytosed (PHK26^+^) and PS^+^ clone 95/3 when added to APCs but not directly to clone 95/3 as compared with IgG control mAbs (Figure 4, A and B, upper panel; and Supplemental Figure 3A). On the other hand, aPD-1 and aPS but not aTim-3 mAbs added to clone 95/3 decreased the frequencies of trogocytosed and PS^+^ clone 95/3 cells as compared with IgG mAbs (Figure 4, A and B, lower panel; and Supplemental Figure 3A). As a control, we performed PD-L1 blockade in DCs or CD8^+^ T cells and found no effect on CD8^+^ T cell trogocytosis (Supplemental Figure 3C). These findings show that Tim-3 blockade acted on Tim-3^+^ APCs but not CD8^+^ T cells to impede trogocytosis in a PS-dependent fashion. In addition, PD-1 blockade reduced PS expression by TA-specific CD8^+^ T cells upon prolonged stimulation with cognate antigen and decreased T cell trogocytosis. To confirm the relevance of these findings, PD-1^+^Tim-3^–^ and PD-1^+^Tim-3^+^ CD8^+^ TILs were sorted from single-cell suspensions obtained from MM, labeled with PKH67, and coincubated 30 minutes with PKH26^+^ autologous CD45^+^CD3^–^ cells and IgG control, aTim-3.18, or aPD-1 mAbs before flow cytometry and ImageStream (Figure 4C and Supplemental Figure 3B). PD-1^+^Tim-3^+^ but not PD-1^+^Tim-3^–^ CD8^+^ TILs acquired membrane fragments from autologous APCs. In this short assay, aTim-3.18 but not aPD-1 mAbs reduced the trogocytosis of PD-1^+^Tim-3^+^ CD8^+^ TILs.

We next evaluated whether early blockade of Tim-3 regulated DC-mediated T cell priming. To this end, DCs prepared from 1 HLA-A2^+^ HD were pulsed with NY-ESO-1 protein, LAGE-1 ORF2 protein as control, HLA-A2–restricted peptide NY-ESO-1 157-165, or cell lysates from 2 melanoma cell lines (NY-ESO-1^+^ MEL 285 or NY-ESO-1^–^ MEL 136) as previously reported (33). In some wells, we added aTim-3 or aPD-L1 mAbs. Cells were washed and added to ELISPOT wells in the presence of NY-ESO-1 157-165–specific T cell clone prior to IFN-γ ELISPOT assay as previously reported (32). Neither Tim-3 blockade nor PD-L1 blockade significantly augmented recognition of NY-ESO-1 protein or NY-ESO-1^+^ melanoma cell lysates by clone 95/3 (Supplemental Figure 3D).

Collectively, our findings show that aTim-3 mAbs act on Tim-3^+^ APCs in a PS-dependent fashion to disrupt the trogocytosis of activated TA-specific CD8^+^ T cells and PD-1^+^Tim-3^+^ CD8^+^ TILs in MM. They also show that PD-1 blockade upon prolonged antigen stimulation decreases PS expression by activated TA-specific CD8^+^ T cells, reducing T cell trogocytosis.

### PD-1 and Tim-3 blockades cooperate to impede the trogocytosis of CD8^+^ TILs in vivo.

**Figure 5:**
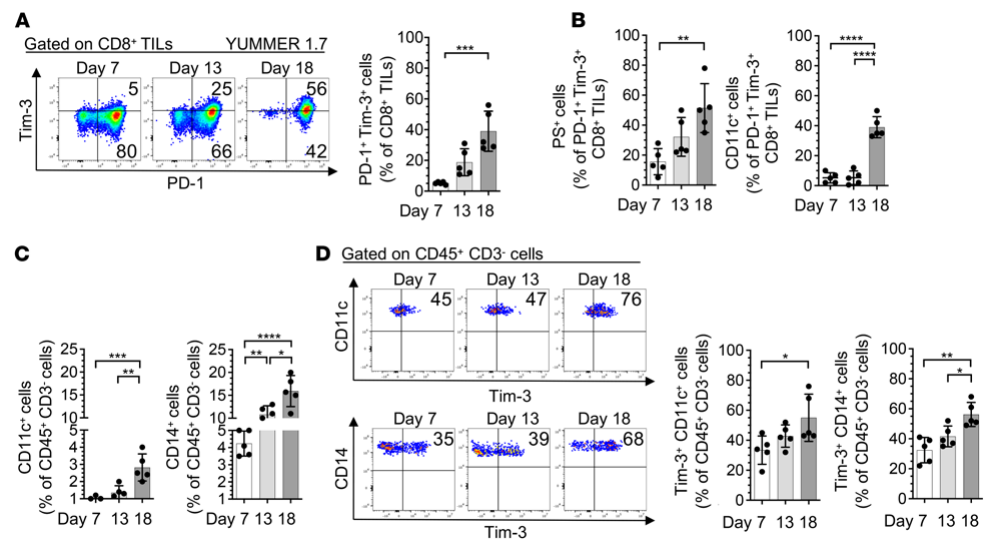
Frequencies of PS^+^, CD11c^+^, and CD14^+^ PD-1^+^Tim-3^+^ CD8^+^ TILs as well as Tim-3^+^ APCs increase in mouse melanoma upon tumor progression. (**A**–**D**) Flow cytometry analysis of YUMMER 1.7 melanoma collected at days 7, 13, and 18 after subcutaneous implantations. (**A**) Representative dot plots (left) and summary data (right) showing frequencies of PD-1^+^Tim-3^+^ CD8^+^ TILs. (**B**) Summary data showing frequencies of PS^+^ (left) and CD11c^+^ (right) PD-1^+^Tim-3^+^ CD8^+^ TILs. (**C**) Summary data showing frequencies of CD11c^+^ (left) and CD14^+^ (right) CD45^+^CD3^–^ cells. (**D**) Representative dot plots (left) and summary data (right) showing frequencies of Tim-3^+^CD11c^+^ and Tim-3^+^CD14^+^ CD45^+^CD3^–^ cells. Results shown are from 1 experiment (*n* = 5), representative of 2 independent experiments. *P* values were obtained by 1-way ANOVA followed by Tukey’s multiple-comparison test (**A**–**D**). *P < 0.05; **P < 0.01; ***P < 0.001; ****P < 0.0001. Data indicate mean ± SD.

**Figure 6:**
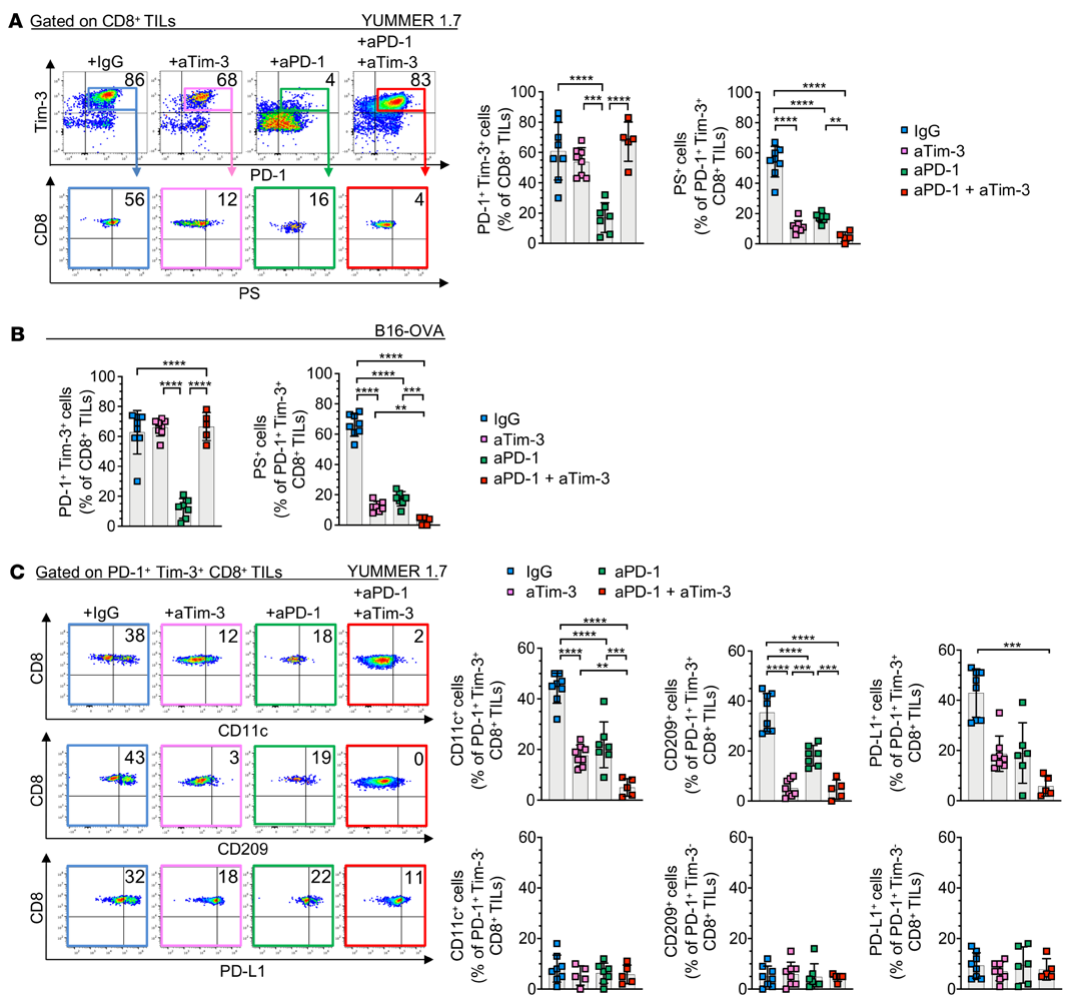
Tim-3 and PD-1 blockades impede the trogocytosis of CD8^+^ TILs in vivo. (**A** and **B**) Representative dot plots (left) and summary data (middle and right) showing frequencies of PD-1^+^Tim-3^+^ and PS^+^PD-1^+^Tim-3^+^ CD8^+^ TILs (day 21) in mice implanted with YUMMER 1.7 (**A**) or B16-OVA (**B**) and treated with indicated mAbs. (**C**) Representative dot plots (left) and summary data (*n* = 5–8, right) showing frequencies of CD11c^+^, CD209^+^, and PD-L1^+^ PD-1^+^Tim-3^–^ and PD-1^+^Tim-3^+^ CD8^+^ TILs (day 21) in mice implanted with YUMMER 1.7 and treated with indicated mAbs. Results shown are from 1 experiment, representative of 3 independent experiments (IgG, *n* = 8; aTim-3, *n* = 8; aPD-1, *n* = 6; aPD-1 + aTim-3, *n* = 5). *P* values were obtained by 1-way ANOVA followed by Tukey’s multiple-comparison test (**A**–**C**) or Kruskal-Wallis test followed by Dunn’s multiple-comparison test (**C**). **P < 0.01; ***P < 0.001; ****P < 0.0001. Data indicate mean ± SD.

We next asked whether trogocytosed CD8^+^ TILs are present in mouse melanoma and whether dual PD-1/Tim-3 blockade impedes the trogocytosis of PD-1^+^Tim-3^+^ CD8^+^ TILs in vivo. To this end, we isolated immune cells from YUMMER 1.7 and B16-OVA melanoma in mice treated with aPD-1 and/or aTim-3 mAbs. In mice with YUMMER 1.7 melanoma, we observed increased frequencies of Tim-3^+^PD-1^+^ CD8^+^ TILs as well as PS^+^, CD11c^+^, and CD14^+^ PD-1^+^Tim-3^+^ CD8^+^ TILs over time (Figure 5, A and B). We also observed increased frequencies of Tim-3^+^ APCs, including CD11c^+^ and CD14^+^CD45^+^CD3^–^ cells in tumors over time (Figure 5, C and D). We then evaluated the effects of Tim-3 and/or PD-1 blockade on the frequencies and trogocytosis of CD8^+^ TILs in mice with YUMMER 1.7 and B16-OVA melanoma. Tim-3 blockade was performed using the RMT3-23 antibody that binds the PS binding site of Tim-3 (Supplemental Figure 4B). We observed higher frequencies of PD-1^+^Tim-3^+^ CD8^+^ TILs in the presence of aTim-3 alone or in combination with aPD-1 as compared with aPD-1 alone (Figure 6, A and B, and Supplemental Figure 4B). We also observed lower frequencies of PS^+^PD-1^+^Tim-3^+^ CD8^+^ TILs upon Tim-3 or PD-1 blockade as compared with IgG control, and these effects were more pronounced in the presence of dual PD-1 and Tim-3 blockade. PD-1^+^Tim-3^+^ CD8^+^ TILs exhibited low transcription factor T cell factor 1 and signaling lymphocyte activation molecule 6 expression (Supplemental Figure 4C). The frequencies of CD11c^+^, CD209^+^, CD14^+^, and PD-L1^+^PD-1^+^Tim-3^+^ CD8^+^ TILs decreased upon single blockade and even more significantly upon dual blockade (Figure 6C and Supplemental Figure 4D).

**Figure 7:**
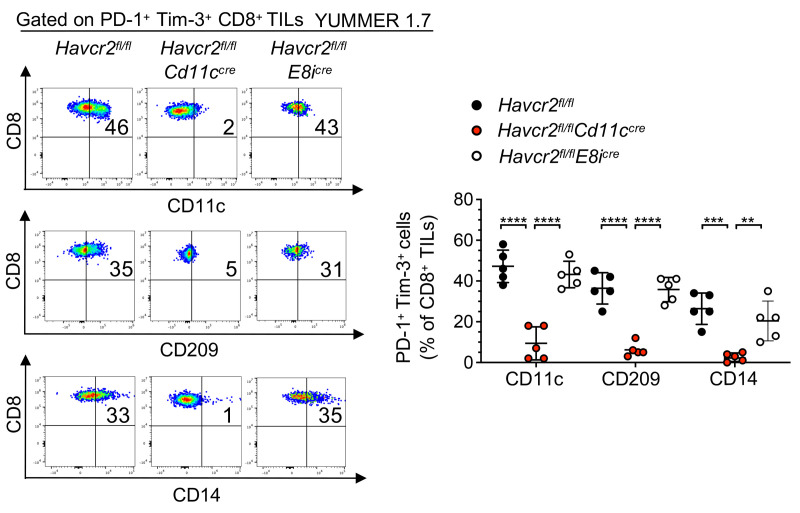
Deleting Tim-3 on DCs but not on CD8^+^ T cells impedes the trogocytosis of CD8^+^ TILs in vivo. Representative flow cytometry dot plots and summary data (*n* = 5, left) showing frequencies of CD11c^+^, CD209^+^, and CD14^+^ PD-1^+^Tim-3^+^ CD8^+^ TILs in *Havcr2^fl/fl^*, *Havcr2^fl/fl^* *Cd11c^cre^*, and *Havcr2^fl/fl^* *E8i^cre^* mice implanted with YUMMER 1.7 (day 21). Results shown are from 1 experiment (*n* = 5), representative of 2 independent experiments. *P* values were obtained from unpaired *t* tests. **P < 0.01; ***P < 0.001; ****P < 0.0001. Data indicate mean ± SD.

To determine whether Tim-3 on DCs regulates the trogocytosis of CD8^+^ TILs, we generated *Havcr2^fl/fl^ E8i^cre^* and *Havcr2^fl/fl^ Cd11c^cre^* mice with Tim-3 deletion in CD8^+^ T cells or CD11c^+^ cells, respectively, and evaluated CD8^+^ T cell trogocytosis in YUMMER 1.7 melanoma (Figure 7). The frequencies of trogocytosed CD8^+^ TILs significantly decreased in *Havcr2^fl/fl^ Cd11c^cre^* but not in *Havcr2^fl/fl^ E8i^cre^* mice, as compared with *Havcr2^fl/fl^* mice, supporting that Tim-3 on DCs but not on CD8^+^ T cells plays a critical role in promoting trogocytosis of PD-1^+^Tim-3^+^ CD8^+^ TILs. Flow cytometry performed from YUMMER 1.7 melanoma surgically excised 21 days after tumor implantation confirmed Tim-3 deletion in CD11c^+^ cells (Supplemental Figure 4E).

Notably, while PD-1 but not Tim-3 blockade significantly decreased tumor burden and increased survival, dual PD-1 and Tim-3 blockade was the most potent therapy at decreasing tumor growth and prolonging survival of mice with YUMMER 1.7 melanoma (Supplemental Figure 5, A–C).

Collectively, these findings show that PD-1^+^Tim-3^+^ CD8^+^ TILs in 2 mouse melanoma models upregulate PS expression as well as myeloid markers, supporting that they spontaneously undergo trogocytosis upon tumor progression. Tumor-infiltrating APCs increased Tim-3 expression upon tumor progression. Further, aPD-1 and aTim-3 mAbs cooperated to impede trogocytosis of PD-1^+^Tim-3^+^ CD8^+^ TILs.

### Trogocytosed CD8^+^ T cells acquire peptide-MHC complexes in a Tim-3–mediated fashion and become the target of fratricide killing.

**Figure 8:**
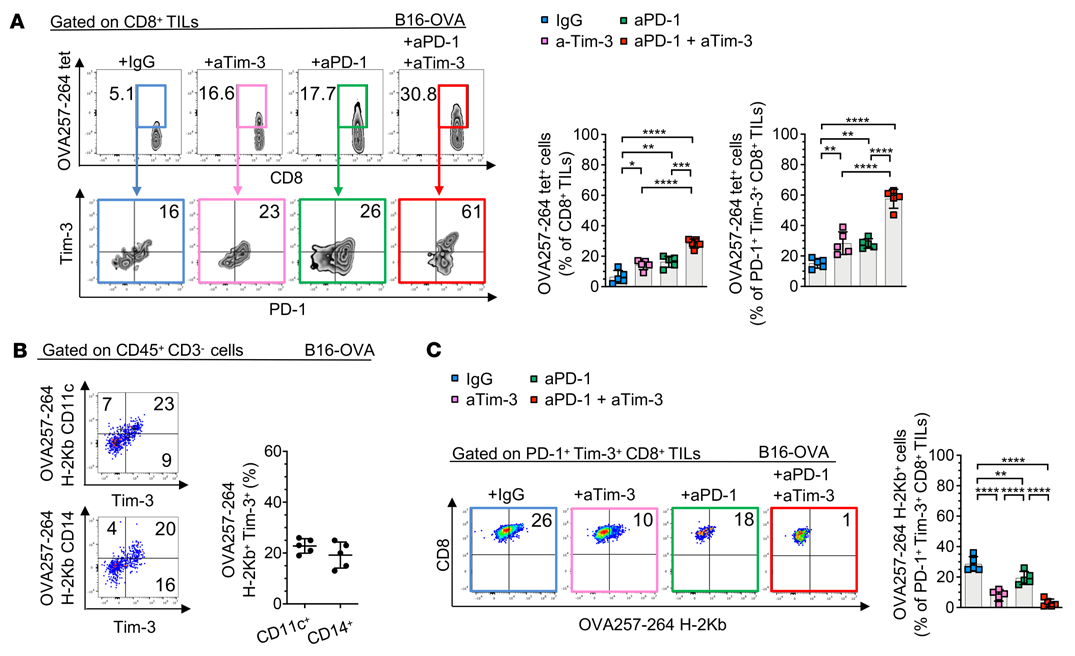
Trogocytosed CD8^+^ T cells acquire peptide-MHC complexes in a Tim-3–mediated fashion. (**A**–**C**) Representative dot plots (left) and summary data (right) showing frequencies of OVA257-264 tet^+^ and PD-1^+^Tim-3^+^tet^+^ CD8^+^ TILs (**A**), peptide-MHC complex (pMHC)/OVA257-264 H-2Kb^+^ Tim-3^+^CD11c^+^ and Tim-3^+^CD14^+^ cells (**B**), or pMHC/OVA257-264-H-2Kb^+^ PD-1^+^Tim-3^+^ CD8^+^ TILs (**C**) in B16-OVA (day 21) treated with indicated mAbs. Results shown are from 1 experiment, representative of 2 independent experiments (*n* = 5). Data are representative of 3 independent experiments. *P* values were obtained by 1-way ANOVA followed by Tukey’s multiple-comparison test (**A** and **C**). *P < 0.05; **P < 0.01; ***P < 0.001; ****P < 0.0001. Data indicate mean ± SD.

We hypothesized that PD-1^+^Tim-3^+^ CD8^+^ TILs acquire peptide-MHC complexes (pMHCs) from APCs through trogocytosis and become the target of tumor-reactive PD-1^+^Tim-3^–^ CD8^+^ T cells. In mice with B16-OVA melanoma, treatment with either aTim-3 or aPD-1 mAbs increased frequencies of OVA257-264 tetramer^+^ (tet^+^) and PD-1^+^Tim-3^+^tet^+^ CD8^+^ TILs as compared with IgG control mAbs, while higher frequencies were observed upon dual PD-1/Tim-3 blockade (Figure 8A and Supplemental Figure 6A). Tumor-infiltrating Tim-3^+^ APCs expressed the OVA257-264/H-2Kb pMHC at day 21 postimplantation (Figure 8B). PD-1^+^Tim-3^+^ CD8^+^ TILs acquired OVA257-264/H-2Kb pMHC, and PD-1 or Tim-3 single blockade decreased the frequencies of OVA257-264/H-2Kb^+^ PD-1^+^Tim-3^+^ CD8^+^ TILs, with further decreased frequencies with combined PD-1/Tim-3 blockade (Figure 8C).

**Figure 9:**
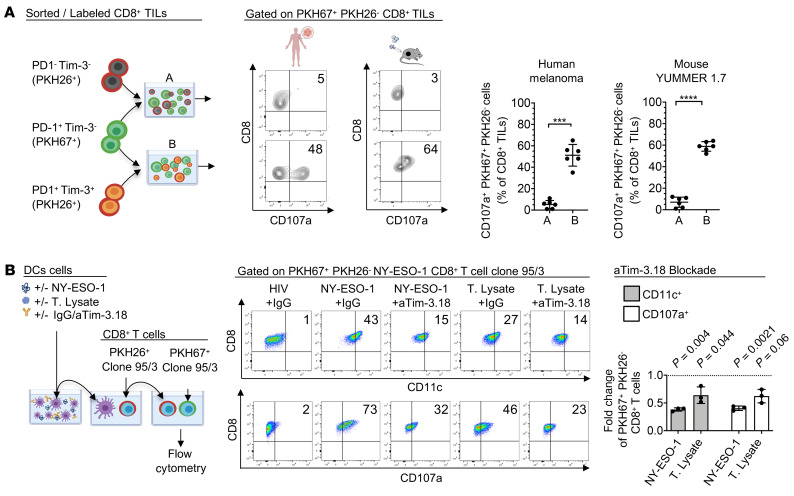
Trogocytosed CD8^+^ T cells become the target of fratricide killing. (**A**) Flow cytometry data showing fratricide killing of PD-1^+^Tim-3^+^ CD8^+^ TILs isolated from human or mouse melanoma. PD-1^+^Tim-3^–^ TILs sorted from human MM or YUMMER 1.7 (day 21 postimplantation) were labeled with PKH67 and cocultured with autologous PKH26-labeled PD-1^–^Tim-3^–^ (group “A” in the figure) or PD-1^+^Tim-3^+^ (group “B” in the figure) CD8^+^ TILs before flow cytometry (left). Representative dot plots (middle) and summary data (right, *n* = 6) showing percentages of PKH67^+^CD107a^+^ CD8^+^ TILs. (**B**) Flow cytometry data showing that Tim-3 blockade impedes fratricide killing of NY-ESO-1–specific CD8^+^ T cells in vitro. DCs obtained from HLA-A2^+^ HDs were pulsed with either tumor lysate (T. Lysate) from an HLA2/NY-ESO-1^+^ melanoma cell line, MEL 285, or NY-ESO-1 157-165 peptide (NY-ESO-1) with aTim-3 or IgG control mAbs, then washed and cocultured with PKH26-labeled clone 95/3. PKH26^+^CD8^+^ T cells were sorted and coincubated with PKH67^+^ clone 95/3 for 6 hours before flow cytometry (left). Representative dot plots (middle) showing percentages of PKH67^+^PKH26^–^CD11c^+^ and PKH67^+^PKH26^–^CD107a^+^ CD8^+^ T cell clones and summary data of frequency fold changes upon aTim-3 as compared with IgG control mAbs (right, *n* = 6). Data are representative of 3 independent experiments. *P* values were obtained by paired *t* test (**A**). *P* values shown in **B** were obtained from paired *t* tests in Supplemental Figure 5B. ***P < 0.001; ****P < 0.0001. Data indicate mean ± SD.

To investigate whether PD-1^+^Tim-3^+^ CD8^+^ TILs acquire pMHCs from APCs in a Tim-3–mediated fashion to become the target of tumor-reactive PD-1^+^Tim-3^–^ CD8^+^ TILs, we sorted PD-1^+^Tim-3^+^ and PD-1^–^Tim-3^–^ CD8^+^ TILs from either human or YUMMER 1.7 melanoma, labeled cells with PKH26, coincubated cells with PKH67-labeled PD-1^+^Tim-3^–^ CD8^+^ TILs, and performed flow cytometry. We observed increased frequencies of CD107a^+^PKH67^+^PKH26^–^ CD8^+^ TILs when PD-1^+^Tim-3^–^ CD8^+^ TILs, but not PD-1^–^Tim-3^–^ CD8^+^ TILs, were coincubated with PD-1^+^Tim-3^+^ CD8^+^ TILs (Figure 9A).

Next, DCs obtained from HLA-A2^+^ HDs were pulsed with either tumor lysates from an HLA-A2/NY-ESO-1^+^ melanoma cell line, MEL 285, or NY-ESO-1 157-165 peptide with or without aTim-3 or IgG control mAbs, then washed and cocultured with PKH26-labeled clone 95/3. PKH26^+^CD8^+^ T cells were sorted and coincubated with PKH67^+^ clone 95/3 for 6 hours before flow cytometry and ImageStream. PKH26^+^ clone 95/3 coincubated with tumor lysates or NY-ESO-1 peptide–pulsed DCs but not with HIV peptide–pulsed DCs expressed the myeloid marker CD11c. In addition, PKH67^+^CD8^+^ T cell clones upregulated CD107a expression in the presence of PKH26^+^CD8^+^ T cell clones previously exposed to peptide-pulsed or tumor lysates, supporting the occurrence of fratricide killing of trogocytosed CD8^+^ T cells. Finally, we observed lower frequencies of CD107a^+^ and CD11c^+^ PKH67^+^PKH26^–^ CD8^+^ T cell clones upon Tim-3 blockade as compared with IgG control mAb treatment (Figure 9B and Supplemental Figure 6, B and C). Together, our findings demonstrate that activated TA-specific CD8^+^ T cells and PD-1^+^Tim-3^+^ CD8^+^ TILs acquire pMHCs from APCs in a Tim-3/PS-dependent fashion. Trogocytosed CD8^+^ T cells become the target of fratricide killing that is counteracted by aTim-3 mAbs.

## Discussion

Here, we show that PD-1^+^Tim-3^+^ CD8^+^ TILs in mouse and human melanoma acquires membrane fragments from tumor-infiltrating Tim-3^+^ APCs through trogocytosis in a Tim-3/PS-mediated fashion. In agreement with previous findings in human and mouse lung cancer (17), Tim-3 was expressed by the majority of APCs in mouse and human melanoma, including monocytes and DCs, and its expression increased upon tumor progression.

Multiple factors in the TME may act synergistically to increase Tim-3 expression by DCs, including VEGF, IL-10, arginase 1, and hypoxia (17, 34). Although Tim-3 can mediate phagocytosis of apoptotic cells and crosspresentation by mouse macrophages and CD8^+^ DCs, the relevance of these findings to cancer immunology remains elusive (35). Preclinical studies suggested that Tim-3 acts in APCs to regulate antitumor immunity through distinct mechanisms. In mouse and human cancer, Tim-3 acts in DCs to suppress nucleic acid–mediated innate immune responses by interfering with HMGB1-mediated activation of nucleic acid systems (17). In mouse mammary tumors treated with paclitaxel, aTim-3 mAbs act in Tim-3^+^ tumor-infiltrating cDCs to promote CXCL9-mediated recruitment of CD8^+^ T cells in a galectin-9–dependent fashion (25). Tim-3 limits activation of the cGAS/STING pathway in intratumor DCs by suppressing extracellular DNA uptake, and the antitumor efficacy of aTim-3 mAb depends on cGAS and STING (24, 25). In addition, Tim-3 deletion in DCs leads to increased accumulation of reactive oxygen species, resulting in NLRP3 inflammasome activation and enhanced antitumor T cell–mediated immunity (26). Inhibition of inflammasome activation, or downstream effector cytokines IL-1β and IL-18, abrogates the protective antitumor immunity observed with Tim-3 deletion in DCs. Our findings add to these preclinical studies to show that in mouse and human melanoma, Tim-3 also acts as a regulator of membrane transfer between Tim-3^+^ APCs in the TME and CD8^+^ TILs in the TME in a PS-mediated fashion. Trogocytosed PD-1^+^Tim-3^+^ CD8^+^ TILs expressed tumor-derived pMHCs acquired from tumor-infiltrating myeloid cells and became the target of fratricide CD8^+^ T cell killing. Tim-3 blockade impeded trogocytosis of PD-1^+^Tim-3^+^ CD8^+^ T cells to prevent their fratricide killing. Progenitor exhausted CD8^+^ T cells play a critical role in mediating potent antitumor immune response to PD-1 blockade in chronic viral infections and in cancers (10, 11). They differentiate into PD-1^+^Tim-3^+^ CD8^+^ TILs, which exhibit potent lytic activity and contribute to antitumor effects in vivo (11, 12). Tim-3 blockade acted primarily in Tim-3^+^ APCs but not CD8^+^ T cells to impede transfer of myeloid membrane-bound antigens, while PD-1 blockade acted in CD8^+^ T cells but not APCs to decrease PS upregulation by TA-specific CD8^+^ T cells. Our findings support that Tim-3 and PD-1 blockades cooperate to disrupt the Tim-3/PS axis in the TME and impede trogocytosis of CD8^+^ TILs in tumors.

Tim-3 deletion in DCs increases crosspresentation to augment antigen-specific CD8^+^ T cell expansion (26). We observed that neither Tim-3 blockade nor PD-L1 blockade significantly augmented recognition of DCs pulsed with NY-ESO-1 protein or NY-ESO-1^+^ melanoma cell lysates by NY-ESO-1–specific CD8^+^ T cell clones, suggesting that Tim-3 blockade does not significantly increase DC crosspresentation capability. In addition to these findings in vitro, the role of Tim-3 blockade in modulating T cell priming in vivo will need to be investigated. The reasons Tim-3 deletion and Tim-3 blockade may differentially regulate antigen presentation by DCs are unclear. One study has raised the hypothesis that endogenous damage-associated molecular patterns in the TME may promote inflammasome activation in tumor-infiltrating Tim-3–deleted DCs. In addition, Tim-3 deletion in DCs may lead to synapse remodeling and stronger TCR signaling than Tim-3 blockade. These hypotheses will need to be investigated in additional experiments in vitro and in vivo.

Although trogocytosis can be bidirectional between conjugated Fcγ receptor–expressing APCs and antibody-bound T cells, ample evidence shows that it is unidirectional for antigen-specific T cells in the presence of unengineered APCs (36, 37). Trogocytosed antigen-specific T cells acquire membrane-associated proteins and pMHCs from APCs and become sensitive to fratricide killing in vitro (38). MHC molecules acquired by trogocytosed antigen-specific T cells are cointernalized with TCRs through endocytosis and localized in endosomes and lysosomes. Our findings show that Tim-3, which is recruited to the immunological synapse upon T cell activation (39, 40), plays a critical role in driving pMHC and membrane-associated protein transfer from Tim-3^+^ APCs to PS^+^ activated T cells both in vitro and in melanoma-bearing mice, regulating T cell trogocytosis and fratricide killing.

Multiple studies have shown the efficacy of dual PD-1 and Tim-3 blockade in established mouse tumors and in early treatment, although the effects on tumor growth appear both model and time dependent (8, 41). In mice recently transplanted with YUMMER 1.7 melanoma, we found that dual PD-1 and Tim-3 blockade exerted superior antitumoral activity and prolonged survival as compared with PD-1 blockade alone. In support of the promising efficacy of dual PD-1 and Tim-3 blockade in cancer, clinical benefits were observed in patients with PD-1–refractory non–small cell lung cancers treated with aPD-1 and aTim-3 mAbs, with a 15% objective response rate (3 out of 19 patients) and with prolonged stable disease in 42% (8 out of 19 patients) in an ongoing phase I/II study (42). Our findings provide mechanistic insights on potential biomarkers of response to dual PD-1 and Tim-3 blockade in patients with cancer.

In summary, our findings uncover a mechanism Tim-3 uses to limit antitumor immunity in mouse and human tumors. Tim-3 on APCs and PS on CD8^+^ TILs cooperated to limit CD8^+^ T cell–mediated antitumor immunity through T cell trogocytosis and fratricide killing. Blockade of Tim-3 and PD-1 disrupted trogocytosis and fratricide killing of antigen-specific CD8^+^ T cells, prolonging their persistence and antitumor reactivity. Dual PD-1 and Tim-3 blockade may prove more efficacious than single blockade in T cell–infiltrated tumors, for which PS and Tim-3 expression by CD8^+^ TILs and APCs, respectively, may serve as potential biomarkers for patient selection.

## Methods

### Human patients and samples

Samples of tumor and peripheral blood mononuclear cells (PBMCs) from 29 patients with stage IV melanoma were obtained under the internal review board–approved protocols UPCI 05–140 and UPCI 96-099, including 22 men and 7 women, ranging from age 31 to 82, recruited from UPMC Hillman Cancer Center. Metastatic sites included skin or soft tissue (20%), lymph nodes (40%), lung (20%), and other visceral locations (20%). The tumor and blood samples were collected before therapy for stage IV melanoma or more than 3 years after the end of IFN adjuvant therapy. Tumor samples were dissociated and enzymatically digested with a tumor dissociation kit (Miltenyi Biotec). Cell suspensions were then aspirated through an 18G needle 10 times and strained through a 70 μm mesh (Miltenyi Biotec) before RBC lysis using ACK lysing buffer (Thermo Fisher Scientific). Human CD45^+^CD3^+^ T lymphocytes and CD45^+^CD3^–^ cells were purified from tumor single-cell suspensions with magnetic separation using REAlease CD3 MicroBead Kit (Miltenyi Biotec). Cells were cultured in complete Iscove’s DMEM (10% human serum, 1% penicillin-streptomycin, 1% l-glutamine, 1% nonessential amino acids [NEAA], and 25 mM HEPES) (Gibco, Thermo Fisher Scientific) at 37°C in a 5% CO_2_ incubator.

### Cells

HLA-A2^+^NY-ESO-1^+^ melanoma cell lines UPCI-MEL-285 and UPCI-MEL-136 were derived from metastatic tumors in-house and cultured in complete RPMI 1640 from Thermo Fisher Scientific (10% fetal bovine serum, 1% penicillin-streptomycin, 1% l-glutamine, 1% NEAA). Tumor-reactive NY-ESO-1–specific CD8^+^ T cell clone 95/3 recognizing the HLA-A2–presented epitope NY-ESO-1 157-165 was generated as previously reported (32), then expanded with allogenic feeder cells in complete Iscove’s DMEM containing PHA (1 μg/mL) (MilliporeSigma) and rhIL-2 (200 IU/mL) (PeproTech). Human DCs were prepared from HLA2^+^ melanoma patients. PBMCs were incubated for 2 hours in a 75 cm^2^ flask before removal of nonadherent cells and 3 rinses with 1× Dulbecco’s PBS (DPBS) (Gibco, Thermo Fisher Scientific). Cells were then cultured for 5 days in AIM V medium (Gibco, Thermo Fisher Scientific) with 1000 UI/mL GM-CSF (PeproTech) and 20 ng/mL IL-4 (PeproTech). Mature DCs were then obtained by stimulating immature DCs with IL-1β/IL-6/TNF-α (PeproTech, 10 ng/mL) for 2 days. To prepare melanoma lysates, cells were lysed in AIM V medium at a concentration of 1 × 10^7^ cells/mL by 5 rapid freeze-thaw cycles. Cell lysates were centrifuged at 18,000*g* at room temperature for 10 minutes. Supernatants were collected and stored at −80°C and then used as previously reported (33). Murine melanoma cell line YUMMER 1.7 was provided by Marcus Bosenberg (Yale School of Medicine) and generated as previously reported (43). B16-OVA was provided by Dario Vignali (University of Pittsburgh). Mouse cell lines and mouse TILs were cultured in DMEM: F12 medium (ATCC) with 10% fetal bovine serum and 1% NEAA (Gibco, Thermo Fisher Scientific) and incubated at 37°C with 5% CO_2_. All cell lines were mycoplasma free as determined by PlasmoTest Mycoplasma Detection kit (InvivoGen).

### Flow cytometry and functional assays

The following conjugated mAbs were used in flow cytometric experiments: human antibodies: CD3 (clone UCHT1, Thermo Fisher Scientific), CD8 (clone RPA-T8-RUO, BD Biosciences), CD45 (clone HI30-RUO, BD Biosciences), CD14 (clone MOP-9-RUO, BD Biosciences), CD11c (clone B-ly6-RUO, BD Biosciences), CD209 (clone DCN46, BD Biosciences), PD-1 (clone MIH4, Invitrogen, Thermo Fisher Scientific), Tim-3 (clone 344823, R&D Systems, Bio-Techne), PD-L1 (clone 29E.2A3, BioLegend), A0201 SLLMWITQC NY-ESO-1 157-165 dextramer (Immudex), CD19 (clone HIB19, BioLegend), CD56 (clone MEM-188, BioLegend), granzyme A (clone CB9, Thermo Fisher Scientific), granzyme B (clone GB11, Thermo Fisher Scientific), CD107a (clone H4A3, BioLegend), perforin (clone δG9-RUO, BD Biosciences), IL-2 (clone MO1-17H12, BioLegend), IFN-γ (clone 4S.B3, BioLegend), and TNF (clone MAb11, BD Pharmingen); mouse antibodies: CD107a (clone 1D4B, Thermo Fisher Scientific), CD209 (clone MMD3, BioLegend), PD-1 (clone RMP1-30, BioLegend), Tim-3 (clone 5D12, BD Biosciences), CD3 (clone 145-2C11, BD Biosciences), CD45 (clone 30-F11, BD Biosciences), CD11c (clone N418, BioLegend), PD-L1 (clone 10F.9G2, BD Biosciences), CD8 (clone 53-6.7, Thermo Fisher Scientific), CD4 (clone GK1.5, BioLegend), CD14 (clone Sa14-2, BioLegend), CD19 (clone 6D5, BioLegend), and NK-1.1 (clone PK136, BioLegend).

For B16-OVA tumors, cells were also stained with MHC tetramer for detection of OVA/SIINFEKL-specific T cells (Immudex) and MHC class I molecule Kb bound to the peptide SIINFEKL (Kb-SIINFEKL) H-2Kb (clone 25-D1.16, BioLegend).

PS/annexin V (BD Biosciences) staining was performed after cell surface staining per the manufacturer’s instructions. Cell viability was assessed using LIVE/DEAD fixable violet kit (Thermo Fisher Scientific) or Zombie NIR (BioLegend).

For intracellular staining, cells were fixed with BD Cytofix/Cytoperm (for intracellular cytokine stains). T cells were incubated with 10 μg/mL brefeldin A, 0.2 μg/mL ionomycin, and 0.5 μg/mL PMA (all from MilliporeSigma) for 6 hours at 37°C followed by staining and fixation. Single-cell tumor suspensions were stained and sorted on a FACSAria flow cytometer, and data were collected using LSR II and FACSDiva software (BD Biosciences), as well as Aurora and SpectroFlo software (Cytek). Analysis was performed using FlowJo Software v10 (BD Biosciences). In some experiments, data were also collected using ImageStreamX MARK II cytometer with INSPIRE Software (Amnis, MilliporeSigma). The flow rate was set at minimum, and the objective magnification was set at 60×. A multi-fluorophore-labeled sample and a series of single-stained cell samples were used to determine accurate laser settings, avoid oversaturation, and calculate compensations. Gradient root mean square and aspect ratio versus area on the bright-field channel were used during acquisition to ensure collection of focused single cells. At least 5 × 10^3^ live cells were acquired per sample. Data analysis was performed using IDEAS Software (Amnis, MilliporeSigma).

### Trogocytosis assays

A total of 10 × 10^6^ cells obtained from melanoma single-cell suspensions were stained with CD45, CD3, CD8, PD-1, and Tim-3 and sorted according to PD-1 and Tim-3 expression under sterile conditions using FACSAria. PD-1^+^Tim-3^–^ and PD-1^+^Tim-3^hi^ CD8^+^ T cells were labeled with PKH67 and then separately coincubated with sorted autologous CD3^–^CD45^+^ (effector-to-target ratio, 1:3) and labeled PKH26 at 37°C for 30 minutes before analysis. After coculture, cells were fixed with 1% of paraformaldehyde for 15 minutes. PKH67- and PKH26-labeled cells were then gently transferred to coverslips and mounted with Fluoroshield supplemented with DAPI (MilliporeSigma) prior to analysis with confocal microscopy (Olympus 1002, original magnification 40×, scale bar: 5 μm). Alternatively, after coculture, CD8^+^ T cells were stained separately with CD11c, CD14, and PD-L1 antibodies prior to flow cytometry analysis with LSR II or ImageStreamX MARK II cytometers. In some experiments, aTim-3.18 (Bristol Myers Squibb, BMS) or aPD-1 (BMS 936558) blocking or IgG4 (DT-1D12-g4P, BMS) isotype-matched control mAbs were added (10 μg/mL). Cells were then stained and analyzed with LSR II or ImageStreamX MARK II cytometers. In some experiments, PHK26-labeled DCs prepared from HLA-A2^+^ HDs or HLA-A2/NY-ESO-1 157-165–specific CD8^+^ T cell clone 95/3, respectively, were incubated in the presence of aTim-3.18, aTim-3.22, aPD-1, aPS (clone 1H6 05-719, MilliporeSigma), aPD-L1 (BMS 936559), or isotype-matched control (DT-1D12-g4P, BMS) mAbs for 1 hour at 37^o^C. Cells were washed with 1× PBS and then cocultured for 5 days (CD8-to-DC ratio, 3:1) in the presence of cognate peptide or irrelevant HIVpol 476-484 peptide (10 mg/mL) prior to flow cytometry analysis.

### Fratricide assays

CD8^+^ TILs obtained from tumor single-cell suspensions were stained and sorted according to PD-1 and Tim-3 expression. PD-1^–^Tim-3^–^ and PD-1^+^Tim-3^+^ subsets were labeled separately with PKH26 whereas PD-1^+^Tim-3^–^ cells were labeled with PKH67 prior to a 6-hour coculture experiment between PKH67^+^ cells and PKH26^+^ cells in the presence of CD107a (human or mouse) antibodies. In other experiments, HLA-A2^+^ DCs were incubated in the presence of either NY-ESO-1 157-165 or HIVpol 476-484 peptide (10 mg/mL), HLA-A2^+^NY-ESO-1^+^ UPCI-MEL 285 freeze-thaw lysates (33), and aTim-3.18 or isotype control mAbs. After 4-hour incubation, DCs were washed 3 times and added to wells with PKH26-labeled NY-ESO-1 157-165–specific clone 95/3 at 37°C. CD8^+^ T cells were then sorted and added to wells in the presence of PKH67-labeled clone 95/3 for 30 minutes at 37°C (ratio, 1:1) for 6 hours prior to flow cytometry for CD107a expression.

#### In vitro stimulation with peptides and ELISPOT assay.

DCs (1 × 10^6^) obtained from 1 HLA-A2^+^ HD were incubated with 1 μM recombinant protein (NY-ESO-1 or LAGE-1 ORF2), cell lysates from 2 melanoma cell lines (NY-ESO-1^+^ MEL 285 or NY-ESO-1^–^ MEL 136, 3 × 10^6^/well), or peptide NY-ESO-1 157-165 for 4 hours at 37°C in the presence of 1 μg/mL LPS (MilliporeSigma) as previously reported (33). In some wells, aTim-3 or aPD-L1 mAbs (10 mg/mL) were added. DCs were subsequently washed 3 times before being added to the CD8^+^ T cell clone (10^3^/well). Spot numbers and spot sizes were determined with computer-assisted video image analysis (Cellular Technologies). Statistical evaluation was performed with 2-tailed *t* test. *P* values less than 0.05 were considered significant.

### Animals and in vivo experiments

Six- to 8-week-old C57BL/6J, *Cd11c^cre^*, and *E8i^cre^* mice were purchased from The Jackson Laboratory. Mice were implanted with YUMMER 1.7 or B16-OVA tumors. Cells were harvested at approximately 60% to 85% confluence on the day of injection, trypsinized with 0.25% trypsin for approximately 2 to 3 minutes before deactivation with complete RPMI 1640, washed twice with sterile 1× HBSS (Life Technologies, Thermo Fisher Scientific), and counted with a Cellometer Auto 2000 counter (Nexcelom Bioscience). A total of 5 × 10^5^ cells were suspended in 100 μL of sterile 1× DPBS and injected subcutaneously into each mouse’s shaved rear flank using a 27G needle (Thermo Fisher Scientific). Mice were monitored at day 6 and every 3 days for the appearance of tumor after injection to begin digital caliper measurements. Two dimensions were taken for calculation of tumor volume according to the equation (W^2^ × L)/2. When indicated, mice were injected intraperitoneally with 10 mg/kg aTim-3 (RMT3-23), aPD-1 (29F.1A12), aPD-1, and aTim-3 mAbs or an isotype-matched control IgG2a (23A) antibody (BioXCell) at days 11, 14, and 17. Mice were sacrificed at day 21. In addition, untreated mice implanted with YUMMER 1.7 were sacrificed at days 7, 13, and 18 prior to tumor resection and flow cytometry. Tumors were harvested and stored on ice in DMEM: F12 medium with 10% of fetal bovine serum and 1% NEAA (Gibco, Thermo Fisher Scientific), then mechanically minced prior to single-cell suspension. For survival experiments, mice bearing tumors of 150 to 200 mm^3^ were randomized into treatment groups and treated with 10 mg/kg aTim-3 (RMT3-23), aPD-1 (29F.1A12), aPD-1, and aTim-3 mAbs or an isotype-matched control IgG2a (23A) antibody by intraperitoneal injection every 4 days for 3 weeks. Animals whose tumors grew larger than 2000 mm^3^ were euthanized. Animals whose tumors became ulcerated prior to progression or complete response were euthanized and removed from the study.

*Havcr2^fl/fl^* mice were created by the University of Pittsburgh Department of Immunology Transgenic and Gene Targeting core (44). Tim-3 conditional knockout mice were generated by crossing *Havcr2^fl/fl^* mice to *Cd11c^cre^* and *E8i^cre^* lines. Deletion efficiency was determined by flow cytometry. YUMMER 1.7 cells (0.5 × 10^6^ cells) were subcutaneously implanted into *Havcr2^fl/fl^* *Cd11c^cre^*, *Havcr2^fl/fl^* *E8i^cre^*, and *Havcr2^fl/fl^* mice as controls. Mice were sacrificed at day 21, and tumors were harvested prior to single-cell suspension and flow cytometry. Mice of both sexes were used throughout the study; sex-matched and age-matched (8–12 weeks) controls were used in individual experiments. Animal experiments were done in accordance with the guidelines of the Institutional Animal Care and Use Committee at the University of Pittsburgh Animal Research Protection Office.

#### scRNA-Seq.

Tumor biopsies from 18 patients were freshly processed into single-cell suspensions. CD45^+^ cells were isolated using FACS and processed using 10x Genomics’ Chromium platform for droplet-based scRNA-Seq with the Chromium Single Cell 5′ Library Construction Kit (v1.0 chemistry, PN-PN-1000006), following the CG000086 user guide. The libraries were sequenced on the Illumina NovaSeq 6000 System with a PE150 configuration to an average depth of 46,000 read pairs/cell. Cell Ranger v4.0.0 (10x Genomics) was used to align the sequenced libraries to the GRCh38-2020-A reference genome with default settings. The resulting gene count matrices were analyzed in R 3.6 using the standard workflow of Seurat v3.2.0 (45). Count log normalization was performed with NormalizeData, which divides the feature counts of each cell by the total counts for that cell, multiplied by 10,000, followed by taking the natural log. To remove dead and/or dying cells, cells with a percentage of genes that map to the mitochondrial genome greater than 10% are removed. Additionally, any cell with unique feature counts less than 200 (empty droplets) or greater than 3000 (multiplets) were removed. To integrate the data and remove batch effects, FindVariableFeatures, FindIntegrationAnchors, and IntegrateData were used with default settings. Clustering to identify different myeloid cell groups was performed in 2 steps. First, clustering was performed to find any myeloid-like cells. Second, the myeloid-like cells were isolated, and another round of clustering was performed to identify cell types. The first round of clustering was performed using FindNeighbors and FindClusters with a resolution of 2. Of the 39 clusters identified, 5 had gene signatures resembling myeloid-like cells (Supplemental Figure 5A and Supplemental Table 2). The second round of clustering was performed with the same methods and a resolution of 1. This produced 12 clusters, 5 of which were identified as monocytes, 2 as cDCs, and 5 as nonmyeloid cells (Supplemental Figure 5, A and B, and Supplemental Table 3). The cDC and monocyte clusters were combined and the cells separated into HAVCR2^+^ (Tim-3) and HAVCR2^–^ populations based upon a normalized expression cutoff of 0.5. Differential gene expression was performed with FindMarkers using default settings. Twenty genes had an adjusted *P* < 0.5. All of these were upregulated in the HAVCR2^+^ population. This gene list was processed using the Metascape web portal (46).

### Crystallography and protein expression and purification

For Tim-3 IgV domain expression, DNA encoding residues 22 to 132 of human Tim-3, or residues 20 to 133 of murine BALB/c residues subcloned into pET47b with N‑terminal 6x‑His followed by 3C cleavage tags, was expressed in *E*. *coli* BL21(DE3) (Thermo Fisher Scientific). Purification and refolding were done following the previously published protocol for murine Tim-3 (19). Inclusion bodies were purified, solubilized with urea, and refolded before affinity purification on Ni Sepharose excel (GE Healthcare, now Cytiva) followed by size-exclusion chromatography (SEC) on a HiLoad 26/600 Superdex 200 pg column (GE Healthcare). For crystallography, Tim-3.18 Fab was expressed by transient transfection into Expi293 cells (Thermo Fisher Scientific). Following 5 to 6 days of transient expression, the supernatant was harvested, filtered, and purified via a C‑terminal 6x-His tag on the heavy chain using Ni Sepharose excel to isolate recombinant protein followed by SEC on either HiLoad 26/600 Superdex 75 pg or 200 pg (GE Healthcare). The antibodies Tim-3.18 and Tim-3.22 were expressed by transient transfection into Expi293 cells and purified via Protein A MabSelect SuRe (GE Healthcare). Human Tim-3-mFc fusion protein was expressed in CHO cells (ATCC) and purified via Protein A MabSelect SuRe followed by SEC. RMT3-23 was purchased from BioXCell. Mouse Tim-3 extracellular domain fused to human Fc was purchased from R&D Systems, Bio-Techne. Crystals of Tim-3.18:hTim-3 IgV complex were grown in hanging drops at 22°C containing 1 μL protein and 1 μL well solution consisting of optimized conditions of 0.1 M MES pH 7.0 and 14.6% to 15.4% PEG 3350 (both from MilliporeSigma). Single crystals were harvested with glycerol as the cryoprotectant and flash frozen in liquid nitrogen. Data collection was conducted at Industrial Macromolecular Crystallography Association-Collaborative Access Team at Advanced Photon Source using Pilatus-6M detector (Dectris). Diffraction images were processed with Global Phasing software and phased using Phaser with VH/VL models of Tim-3.18 (MOE, Chemical Computing Group) with Fab constant regions from PDB 4NZU and hTim-3 IgV from PDB 5F71. Multiple rounds of refinement were done using Refmac (47), Coot (47), and Phenix (48).

### Yeast display epitope mapping

A 2-micron plasmid encoding human or murine BALB/c Tim-3 extracellular domain inserted upstream of a myc-Sag1 element was created to display Tim-3 on yeast strain BJ5464 (ATCC). GeneMorph II random mutagenesis kit (Agilent) was used with the low mutation rate protocol supplied by the manufacturer. Antigen expression was assessed with mouse anti-myc (clone 9E10, Thermo Fisher Scientific) and polyclonal goat anti-mouse–APC secondary (Thermo Fisher Scientific). Yeast cells were gated to isolate singlets with positive antigen expression and negative binding by aTim-3 Fab and subsequently expanded. An unselected yeast population was included as a reference. After 2 rounds of selection, yeast plasmids were isolated using Zymoprep Yeast Plasmid Miniprep II (Zymo Research), and DNA libraries were created using the Nextera XT preparation kit (Illumina) to generate fragments followed by MiSeq sequencing.

To determine epitope positions, frequencies of amino acid mutations were counted for each position, including a reference sample from an unselected yeast population. Mutation frequencies for each library were normalized using the median of geometric mean ratios across samples (49); positions causing loss in binding were denoted having log_2_ enrichment ≥ 2 relative to median mutation frequency within the sample. Positions were excluded from the putative epitopes if they were involved in a disulfide bond or buried in the protein interior.

### Determination of binding kinetics and affinities via surface plasmon resonance

Binding kinetics were measured with a Biacore T200 surface plasmon resonance instrument (GE Healthcare) at 37°C. The running buffer was 10 mM HEPES, 150 mM NaCl, and 0.05% (v/v) Tween 20 at pH 7.4 supplemented with 1 g/L BSA. Binding of human Tim-3 to aTim-3.18 and aTim-3.22 was measured in a single-cycle kinetics measurement. The antibodies were captured on a CM4 chip with preimmobilized anti-human IgG Fc capture pAb (Southern Biotech, catalog number 2081-01), and human Tim-3 was injected as an analyte in increasing concentrations of a 5-membered, 5-fold dilution series with 1 μM top concentration, followed by a dissociation phase in running buffer and regeneration of the capture surface with 75 mM phosphoric acid. Each antibody was measured in duplicate in different cycles and on different flow cells. Binding of mouse Tim-3 to RMT3-23 was measured in a multicycle kinetics measurement. RMT3-23 was captured in increasing surface densities on flow cells 2, 3, and 4 of a CM4 chip with preimmobilized anti-murine IgG capture pAb (Cytiva catalog number BR100838). Mouse Tim-3 was injected as an analyte in a 5-membered, 3-fold dilution series with 730 nM top concentration and a duplicate injection at 240 nM. Each analyte injection was followed by a dissociation phase in running buffer and regeneration of the capture surface with 10 mM glycine (MilliporeSigma) pH 1.7. All data were double referenced using flow cell 1 and a buffer injection before fitting to a 1:1 Langmuir binding model with mass transport using Biacore T200 Insight Evaluation Software 3.1 (GE Healthcare).

### Cell-free PS blocking assay

PS liposomes were produced by transferring 2 mg of PS into a borosilicate glass tube and dried from the solvent before adding 1 mL of PBS. The tube was then vortexed and sonicated gently by immersing a sonotrode microtip and using the lowest power setting in 0.5 second pulses, until a cloudy emulsion formed (approximately 15 seconds). The resulting liposomes in PBS were vortexed again and extruded by passing 11 times through an extruder (LiposoFast from Avestin) using a membrane with 100 nm pore size. An Octet HTX instrument (ForteBio) was used to test if aTim-3 mAbs could directly block the interaction of PS liposomes with Tim-3. The assay was performed at 25°C in PBS pH 7.4 supplemented with 1 g/L BSA. Human Tim-3–mouse Fc fusion protein was captured on anti-mouse Fc tips. Mouse Tim-3–human Fc fusion protein was captured on anti-human Fc tips. Captured Tim-3 was first saturated with an antibody (aTim-3.18, aTim-3.22, RMT3-23, or nonblocking anti-mouse aTim-3 control mAb), and then binding of PS liposomes was tested. The observed PS binding signal was normalized to the highest binding response in the assay.

### Statistics

Statistical analyses were performed in Prism Software (GraphPad). The normality of each variable was evaluated using the Shapiro-Wilk test. For normally distributed data, the comparison between 2 groups of data was performed using unpaired or paired 2-tailed *t* tests, and the comparison between multiple groups of data was performed using ordinary or 1-way ANOVA followed by Tukey’s or repeated measures 1-way ANOVA followed by Holm-Šídák (paired data) multiple comparisons test (all conditions). When data were not normally distributed, 2 paired groups of data were compared with Wilcoxon’s matched pairs signed-rank tests, and multiple groups of data were compared with Kruskal-Wallis test followed by Dunn’s multiple tests (unpaired data) or Friedman’s test followed by Dunn’s multiple tests (paired data). Linear regressions were evaluated with Pearson’s correlation tests. Differences between groups in tumor volume were analyzed by Mann-Whitney *U* test. All experimental data are presented as mean ± SD or mean ± SEM. Significant differences (*P* < 0.05) were indicated for each figure and defined with *P < 0.05; **P < 0.01; ***P < 0.001; ****P < 0.0001.

### Study approval

Samples from patients were obtained after receipt of written informed consent under the internal review board–approved protocols UPCI 05–140 and UPCI 96-099 at UPMC Hillman Cancer Center. Animal experiments were done with the approval of the Institutional Animal Care and Use Committee at the University of Pittsburgh Animal Research Protection Office.

## Author contributions

OP and HMZ conceived the study. OP, RMM, HB, BZ, ACA, VKK, LPK, SCW, MB, AJK, XMS, PS, and HMZ designed methodology. OP, RM, JMC, HB, DD, QD, TT, WG, SRC, RD, AL, KK, BZ, MK, RA, IR, SZ, CS, JMK, PS, and HMZ performed experiments, data collection, and data analysis. AK, AR, SMW, MH, CB, XD, XMS, and PS performed antibody, crystallographic, and binding experiments. HMZ acquired funding. OP, RD, and HMZ performed project administration. PS and HMZ supervised the study. OP, RM, XMS, PS, and HMZ wrote the original draft. OP, RM, JMC, XMS, PS, and HMZ reviewed and edited the draft.

## Supplementary Material

Supplemental data

## Version 1 03/22/2022

In-Press Preview

## Version 2 05/02/2022

Electronic publication

## References

[1] Blackburn SD (2009). Coregulation of CD8^+^ T cell exhaustion by multiple inhibitory receptors during chronic viral infection. Nat Immunol.

[2] Barber DL (2006). Restoring function in exhausted CD8 T cells during chronic viral infection. Nature.

[3] Topalian SL (2012). Safety, activity, and immune correlates of anti-PD-1 antibody in cancer. N Engl J Med.

[4] Hamid O (2013). Safety and tumor responses with lambrolizumab (anti-PD-1) in melanoma. N Engl J Med.

[5] Larkin J (2015). Combined nivolumab and ipilimumab or monotherapy in untreated melanoma. N Engl J Med.

[6] Zarour HM (2016). Reversing T-cell dysfunction and exhaustion in cancer. Clin Cancer Res.

[7] Zhu C (2005). The Tim-3 ligand galectin-9 negatively regulates T helper type 1 immunity. Nat Immunol.

[8] Sakuishi K (2010). Targeting Tim-3 and PD-1 pathways to reverse T cell exhaustion and restore anti-tumor immunity. J Exp Med.

[9] Fourcade J (2010). Upregulation of Tim-3 and PD-1 expression is associated with tumor antigen-specific CD8+ T cell dysfunction in melanoma patients. J Exp Med.

[10] Im SJ (2016). Defining CD8+ T cells that provide the proliferative burst after PD-1 therapy. Nature.

[11] Miller BC (2019). Subsets of exhausted CD8(+) T cells differentially mediate tumor control and respond to checkpoint blockade. Nat Immunol.

[12] Paley MA (2012). Progenitor and terminal subsets of CD8^+^ T cells cooperate to contain chronic viral infection. Science.

[13] Gros A (2014). PD-1 identifies the patient-specific CD8^+^ tumor-reactive repertoire infiltrating human tumors. J Clin Invest.

[14] Ding QQ (2020). Targeting novel inhibitory receptors in cancer immunotherapy. Semin Immunol.

[15] Wolf Y (2020). TIM3 comes of age as an inhibitory receptor. Nat Rev Immunol.

[16] Huang YH (2015). CEACAM1 regulates TIM-3-mediated tolerance and exhaustion. Nature.

[17] Chiba S (2012). Tumor-infiltrating DCs suppress nucleic acid-mediated innate immune responses through interactions between the receptor TIM-3 and the alarmin HMGB1. Nat Immunol.

[18] Cao E (2007). T cell immunoglobulin mucin-3 crystal structure reveals a galectin-9-independent ligand-binding surface. Immunity.

[19] DeKruyff RH (2010). T cell/transmembrane, Ig, and mucin-3 allelic variants differentially recognize phosphatidylserine and mediate phagocytosis of apoptotic cells. J Immunol.

[20] Anderson AC (2007). Promotion of tissue inflammation by the immune receptor Tim-3 expressed on innate immune cells. Science.

[21] Fischer K (2006). Antigen recognition induces phosphatidylserine exposure on the cell surface of human CD8+ T cells. Blood.

[22] Rudd-Schmidt JA (2019). Lipid order and charge protect killer T cells from accidental death. Nat Commun.

[23] Chow A (2021). Tim-4^+^ cavity-resident macrophages impair anti-tumor CD8^+^ T cell immunity. Cancer Cell.

[24] de Mingo Pulido A (2021). The inhibitory receptor TIM-3 limits activation of the cGAS-STING pathway in intra-tumoral dendritic cells by suppressing extracellular DNA uptake. Immunity.

[25] de Mingo Pulido A (2018). TIM-3 regulates CD103(+) dendritic cell function and response to chemotherapy in breast cancer. Cancer Cell.

[26] Dixon KO (2021). TIM-3 restrains anti-tumour immunity by regulating inflammasome activation. Nature.

[27] Eisenberg G (2013). Imprinting of lymphocytes with melanoma antigens acquired by trogocytosis facilitates identification of tumor-reactive T cells. J Immunol.

[28] Gary R (2012). Antigen-specific transfer of functional programmed death ligand 1 from human APCs onto CD8+ T cells via trogocytosis. J Immunol.

[29] Riond J (2007). Capture of membrane components via trogocytosis occurs in vivo during both dendritic cells and target cells encounter by CD8(+) T cells. Scand J Immunol.

[30] Joly E, Hudrisier D (2003). What is trogocytosis and what is its purpose?. Nat Immunol.

[31] Freeman GJ (2010). TIM genes: a family of cell surface phosphatidylserine receptors that regulate innate and adaptive immunity. Immunol Rev.

[32] Fourcade J (2008). Immunization with analog peptide in combination with CpG and montanide expands tumor antigen-specific CD8+ T cells in melanoma patients. J Immunother.

[33] Kudela P (2011). Epitope hierarchy of spontaneous CD4+ T cell responses to LAGE-1. J Immunol.

[34] Koh HS (2015). The HIF-1/glial TIM-3 axis controls inflammation-associated brain damage under hypoxia. Nat Commun.

[35] Nakayama M (2009). Tim-3 mediates phagocytosis of apoptotic cells and cross-presentation. Blood.

[36] Daubeuf S (2010). The direction of plasma membrane exchange between lymphocytes and accessory cells by trogocytosis is influenced by the nature of the accessory cell. J Immunol.

[37] Li G (2019). T cell antigen discovery via trogocytosis. Nat Methods.

[38] Huang JF (1999). TCR-Mediated internalization of peptide-MHC complexes acquired by T cells. Science.

[39] Clayton KL (2014). T cell Ig and mucin domain-containing protein 3 is recruited to the immune synapse, disrupts stable synapse formation, and associates with receptor phosphatases. J Immunol.

[40] Kataoka S (2021). The costimulatory activity of Tim-3 requires Akt and MAPK signaling and its recruitment to the immune synapse. Sci Signal.

[41] Ngiow SF (2011). Prospects for TIM3-targeted antitumor immunotherapy. Cancer Res.

[42] Davar D (2018). A phase 1 study of TSR-022, an anti-TIM-3 monoclonal antibody, in combination with TSR-042 (anti-PD-1) in patients with colorectal cancer and post-PD-1 NSCLC and melanoma. J Immunother Cancer.

[43] Meeth K (2016). The YUMM lines: a series of congenic mouse melanoma cell lines with defined genetic alterations. Pigment Cell Melanoma Res.

[44] Banerjee H (2021). Expression of Tim-3 drives phenotypic and functional changes in Treg cells in secondary lymphoid organs and the tumor microenvironment. Cell Rep.

[45] Stuart T (2019). Comprehensive integration of single-cell data. Cell.

[46] Zhou Y (2019). Metascape provides a biologist-oriented resource for the analysis of systems-level datasets. Nat Commun.

[47] Emsley P (2010). Features and development of Coot. Acta Crystallogr D Biol Crystallogr.

[48] Liebschner D (2019). Macromolecular structure determination using X-rays, neutrons and electrons: recent developments in Phenix. Acta Crystallogr D Struct Biol.

[49] Anders S, Huber W (2010). Differential expression analysis for sequence count data. Genome Biol.

