# Supplementary material for: Tim-3 mediates T cell trogocytosis to limit antitumor immunity
Source: J Clin Invest. 2022 May 2;132(9):e152864. doi: 10.1172/JCI152864 (PMC9057587; doi:10.1172/JCI152864)
Supplement: Supplemental data [file jci-132-152864-s010.pdf]

Supplemental Figure Legends

Fig. S1. PD-1$^+$Tim-3^{high} CD8$^+$ TILs produce less cytokines but exhibit higher lytic capacities than PD-1$^+$Tim-3$^-$ and PD-1-Tim-3$^-$ CD8$^+$ TILs in metastatic melanoma.

(A) Representative dot plots for one metastatic melanoma patient showing expression of PD-1 and Tim-3 on CD8$^+$ TILs (PD-1$^+$ Tim-3^{high} CD8$^+$ T cells in red gate). (B) Summary data (n = 10) showing percentages of cytokine (INFγ, TNF, IL-2) and granzyme (A and B) producing PD-1$^-$Tim-3$^-$, PD-1$^+$Tim-3$^-$, and PD-1$^+$Tim-3^{high} CD8$^+$TILs in metastatic melanoma.
(C) Dot plots from one representative metastatic melanoma showing frequencies of perforin, CD107a$^+$ and PS$^+$ CD8$^+$ TILs according to PD-1 and Tim-3 expression.
(D) Representative dot plots for one metastatic melanoma and summary data (n = 10) showing frequencies of CD209$^+$ CD8$^+$ TILs according to PD-1 and Tim-3 expression.
(E) Summary data showing frequencies of CD11c$^+$ and CD14$^+$ CD45$^+$CD3$^-$ cells in metastatic melanoma (n = 8). (F) Representative flow cytometry dot plots and summary data (n =10, left) evaluating the expression of active caspase-3 and PS by PD-1$^+$ Tim-3$^+$ CD8$^+$ TILs. P-values were obtained by one-way ANOVA followed by Holm-Šídák multiple comparison test and Friedman test followed by Dunn's multiple comparison test (B, F), Wilcoxon matched-pairs signed rank test (D). *P < 0.05; **P < 0.01; ***P < 0.001; ****P < 0.0001. Data indicate mean ± SD.

Fig. S2. Identification of tumor-infiltrating myeloid cell clusters using scRNA-seq.

(A) scRNA-seq data showing a UMAP projection of five myeloid clusters identified during the first clustering step. The bulk of myeloid cells clustered near each other on this

initial UMAP. (B) scRNA-seq data showing myeloid clusters, with cDC and monocyte cells identified and grouped. (C) Expression of common monocyte and cDC markers across UMAP projected cells.

Fig. S3. Tim-3 blockade acts in APCs in a PS-mediated fashion and cooperates with PD-1 blockade to decrease trogocytosis of tumor antigen-specific CD8^{+} T cells.

(A) Summary data showing frequencies of PKH26^{+} and PS^{+} NY-ESO-1-specific clone 95/3 in the presence of HLA-A2^{+} DCs pulsed with cognate peptide and indicated mAbs. Blocking mAbs were added to wells containing DCs before washing and co-incubation with CD8^{+} T cell clones (DC blockade, left), or CD8^{+} T cell clones before washing and co-incubation with HLA-A2^{+} DCs (CD8 Blockade, right). Data are representative of three independent experiments. (B) Summary data showing frequencies of PKH26^{+}PD-1^{+}Tim-3^{+} CD8^{+} TILs co-incubated for 30 min with PKH26^{+}CD45^{+}CD3^{-} cells isolated from metastatic melanoma in the presence of mAbs before flow cytometry (n = 5). (C) Representative flow cytometry dot plots (right) and summary data (left) evaluating PKH26 expression by CD8^{+} T cell clone 95/3 after DC blockade (C, upper) or CD8 Blockade (C, lower). Data shown are representative of three independent experiments. (D) ELISPOT assay with DCs pulsed with proteins, peptides or melanoma cell lysates +/- aTim-3 or aPD-L1 mAbs in the presence of NY-ESO-1-specific CD8^{+} T cell clone 95/3. Spots were developed and counted by computer-assisted video image analysis. Each bar represents the mean spot number of triplicates (± SD) with 1×10^{3} CD8^{+} T cells initially seeded per well. One representative experiment of at least three performed is depicted. P-values were calculated using paired t-tests (A, B and C). n.s. = non-significant (C).

Fig. S4. Dual PD-1 and Tim-3 blockade impedes trogocytosis of $CD8^+$ TILs in mice with Yummer 1.7 or B16-OVA melanoma. (A) Yeast epitope display (left) and cell-free PS binding assay (right) of aTim-3 RMT3-23. Epitope mapping of RMT3-23 on mouse Tim-3 (magenta) shows it binds to the PS binding site (orange sphere). Cell-free PS binding assay shows relative binding of PS liposomes to Tim-3 saturated with RMT3-23 or non-blocking control Ab. (B) Representative dot plots showing frequencies of PD-1^+Tim-3^+ $CD8^+$ TILs (upper) and PS^+PD-1^+Tim-3^+ $CD8^+$ TILs (lower) in B16-OVA tumors (day 21) treated with indicated mAbs. (C) Representative dot plots (left) and summary data (right, n = 5) showing frequenciesof $Tcf1^+$ and $Slamf6^+$ $PD1^+$Tim-3^+ $CD8^+$ TILs in B16-OVA tumors treated with indicated mAbs. (D) Representative dot plots and summary data (n = 5–8) showing frequencies of $CD14^+$PD-1^+Tim-3^+ $CD8^+$ T cells (left and right) and $CD14^+$ PD-1^+Tim-3^- $CD8^+$ TILs (right) in mice implanted with YUMMER 1.7 (day 21) treated with indicated mAbs. (E) Representative flow cytometry dot plots and summary data (n=5, left) evaluating Tim-3 expression by *Havcr2$^{fl/fl}$* and *Havcr2$^{fl/fl}$Cd11c^{cre}* $CD11c^+$ $CD45^+$ $CD3^-$ TILs in YUMMER1.7 (day 21). Results shown are from one experiment, representative of two independent experiments. P-values were calculated using one-way ANOVA followed by Tukey's multiple comparison test (D). $^*P < 0.05$; $^{**}P < 0.01$; $^{***}P < 0.001$; $^{****}P < 0.0001$. Data indicate mean ± SD.

Fig. S5. Dual PD-1 and Tim-3 blockade impedes tumor growth and prolongs survival of mice with YUMMER 1.7 melanoma. (A) Growth curves of YUMMER 1.7 mouse tumors treated with aTim-3 (n = 13), aPD-1 (n = 14), or both mAbs, compared to isotype IgG control. Tumor growth curves are the summary of two independent experiments (n = 11–14). (B) Survival was monitored, and mice were euthanized when tumors reached $2000 mm^3$. Results

shown are from one experiment (n = 5-7), representative of two independent experiments. (D) Individual tumor growth curves of YUMMER 1.7 melanoma treated with indicated mAbs. *P*-values were obtained from Mann-Whitney U test. *P < 0.05; **P < 0.01; ***P < 0.001; ****P < 0.0001. Data indicate mean ± SD.

Fig. S6. Gating strategy of tetramer-positive cells in B16-OVA melanoma and evaluation of trogocytosed NY-ESO-1-specific $CD8^+$ T cell clone in the presence of HLA-$A2^+$ DCs pulsed with cognate antigen and Tim-3 blockade. (A) Flow cytometry gating strategy for H-2kb/OVA257-264 tet^+ $CD8^+$ T cells in B16-OVA tumors. (B) Summary data showing frequencies of $PKH67^+$ $PKH26^-$ $CD11c^+$ and $PKH67^+$ $PKH26^-$ $CD107a^+$ NY-ESO-1-specific $CD8^+$ T cell clone 95/3. DCs obtained from HLA-$A2^+$ healthy donors were pulsed with either tumor lysate (T. Lysate) from an HLA-A2/NYESO-1^+ melanoma cell line MEL 285 or NY-ESO-1 157-165 peptide (NY-ESO-1) ± aTim-3 or IgG control mAbs, then washed and co-cultured with PKH26-labelled clone 95/3. $PKH26^+$ $CD8^+$ T cells were sorted and co-incubated with $PKH67^+$ clone 95/3 for 6 hours before flow cytometry (left). Data are representative of three independents experiments. (C) Representative ImageStream analysis showing cell surface expression of CD11c, CD107a, and PKH26 by $PKH67^+CD8^+$ T cell clone 95/3 with aTim-3 or IgG control mAbs. P-values were calculated using paired t-tests (B). *P < 0.05; **P < 0.01; ***P < 0.001; ****P < 0.0001.

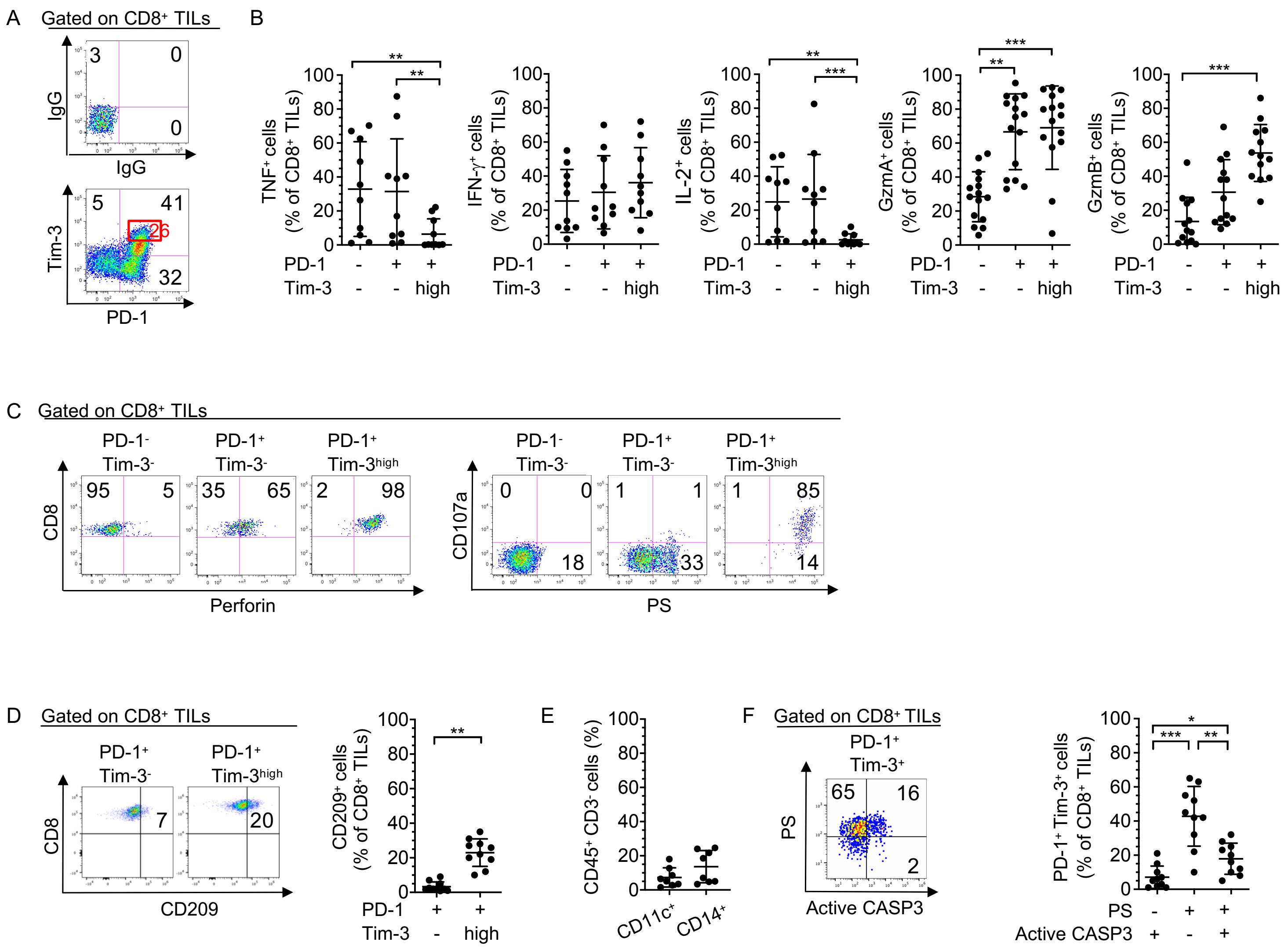

A
Gated on CD8+ TILs
IgG
IgG
Tim-3
PD-1
B
TNF+ cells (% of CD8+ TILs)
IFN-γ+ cells (% of CD8+ TILs)
IL-2+ cells (% of CD8+ TILs)
GzmA+ cells (% of CD8+ TILs)
GzmB+ cells (% of CD8+ TILs)
PD-1
Tim-3
high
C
Gated on CD8+ TILs
PD-1- Tim-3-
PD-1+ Tim-3-
PD-1+ Tim-3high
CD8
Perforin
CD107a
PS
D
Gated on CD8+ TILs
CD8
CD209
CD209+ cells (% of CD8+ TILs)
E
CD45+ CD3- cells (%)
CD11c+
CD14+
F
Gated on CD8+ TILs
PD-1+ Tim-3+
PS
Active CASP3
PD-1+ Tim-3+ cells (% of CD8+ TILs)

Figure S1

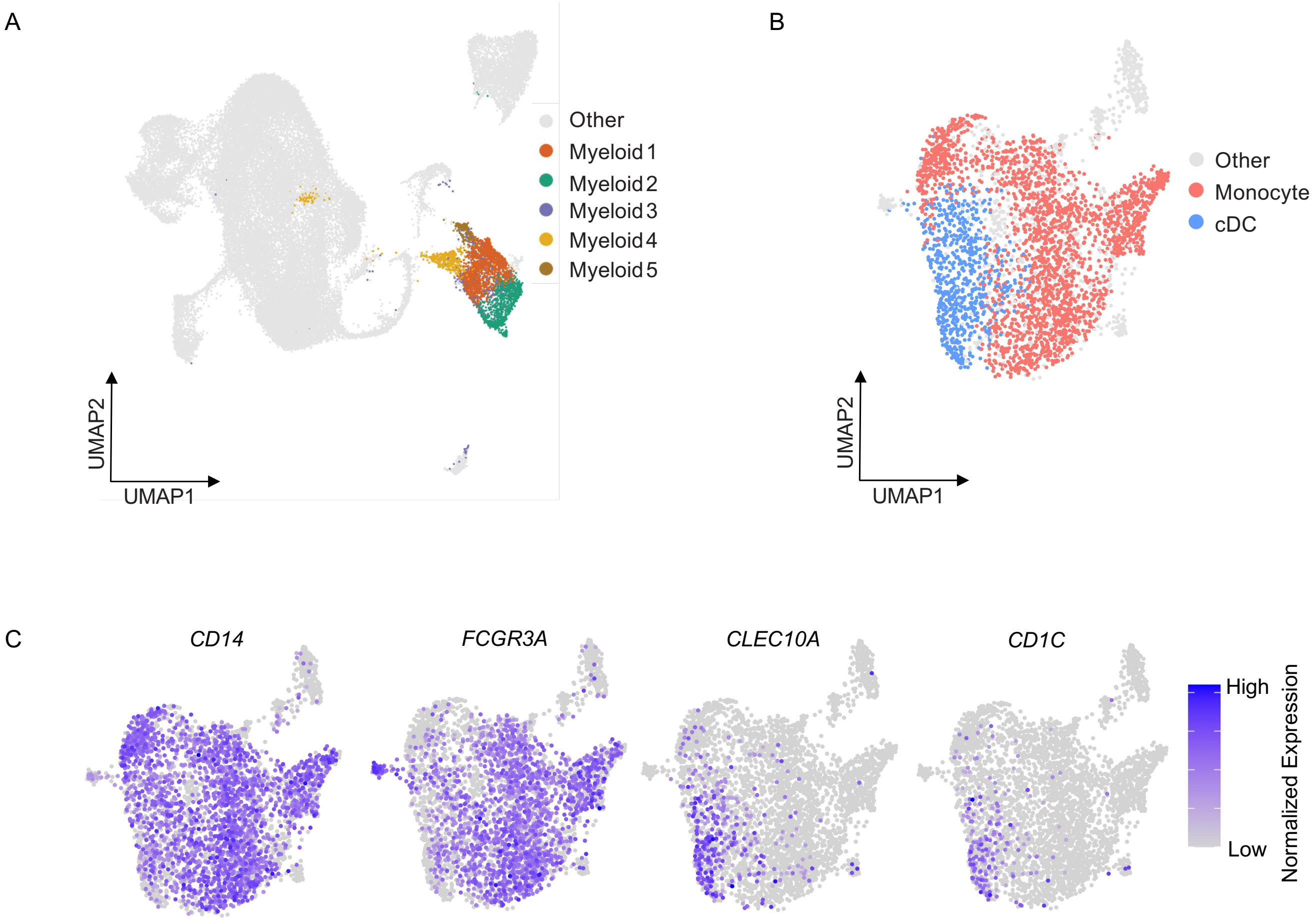
A
Other
Myeloid 1
Myeloid 2
Myeloid 3
Myeloid 4
Myeloid 5
UMAP2
UMAP1
B
Other
Monocyte
cDC
UMAP2
UMAP1
C
CD14
FCGR3A
CLEC10A
CD1C
High
Low
Normalized Expression

Figure S2

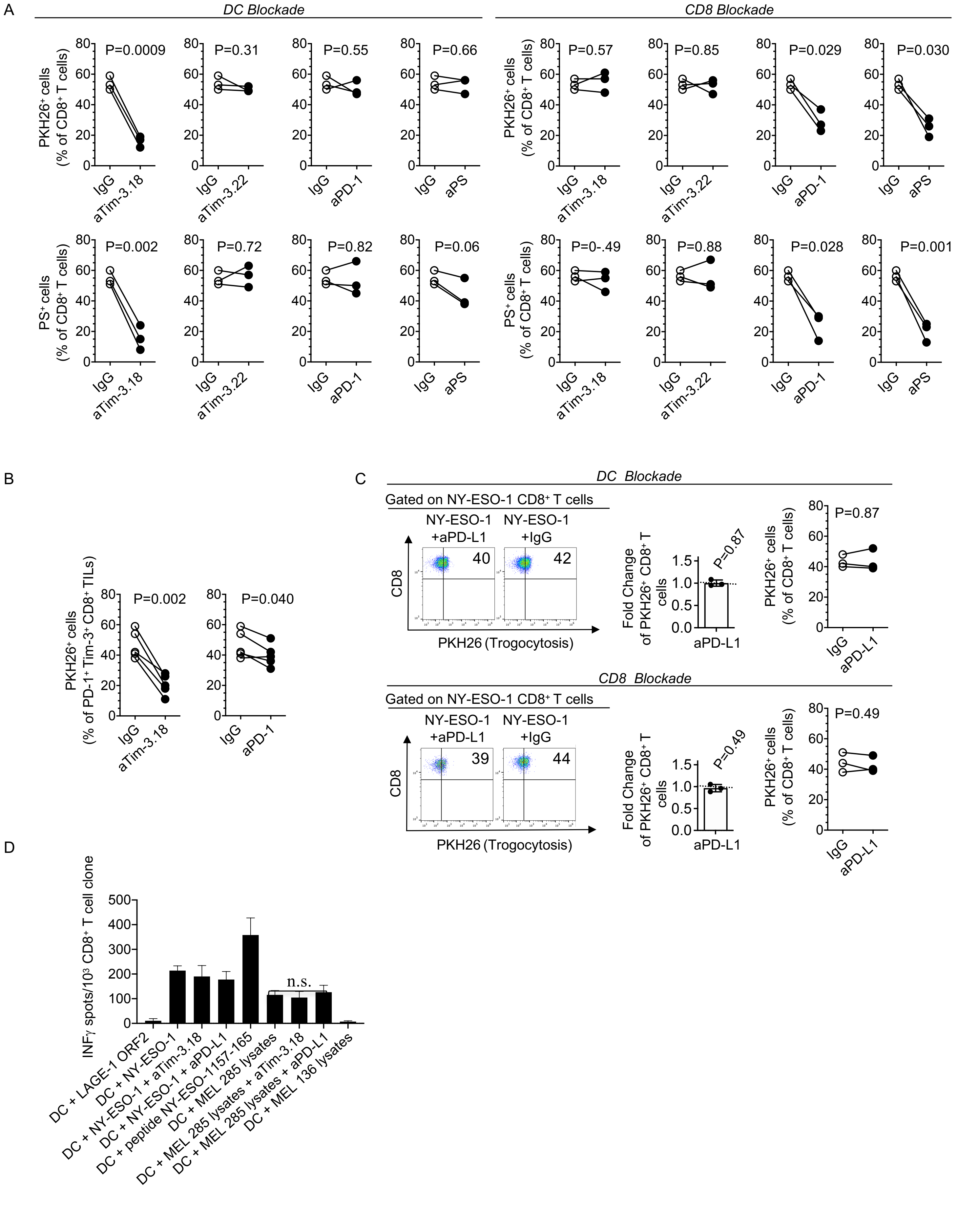
A
DC Blockade
CD8 Blockade
PKH26⁺ cells (% of CD8⁺ T cells)
P=0.0009
P=0.31
P=0.55
P=0.66
P=0.57
P=0.85
P=0.029
P=0.030
IgG
aTim-3.18
aTim-3.22
aPD-1
aPS
PS⁺ cells (% of CD8⁺ T cells)
P=0.002
P=0.72
P=0.82
P=0.06
P=0-.49
P=0.88
P=0.028
P=0.001
B
PKH26⁺ cells (% of PD-1⁺ Tim-3⁺ CD8⁺ TILs)
P=0.002
P=0.040
C
DC Blockade
Gated on NY-ESO-1 CD8⁺ T cells
NY-ESO-1 +aPD-L1
NY-ESO-1 +IgG
40
42
CD8
PKH26 (Trogocytosis)
Fold Change of PKH26⁺ CD8⁺ T cells
P=0.87
aPD-L1
PKH26⁺ cells (% of CD8⁺ T cells)
P=0.87
CD8 Blockade
39
44
P=0.49
P=0.49
D
INFγ spots/10³ CD8⁺ T cell clone
n.s.
DC + LAGE-1 ORF2
DC + NY-ESO-1
DC + NY-ESO-1 + aTim-3.18
DC + NY-ESO-1 + aPD-L1
DC + peptide NY-ESO-1157-165
DC + MEL 285 lysates
DC + MEL 285 lysates + aTim-3.18
DC + MEL 285 lysates + aPD-L1
DC + MEL 136 lysates

Figure S3

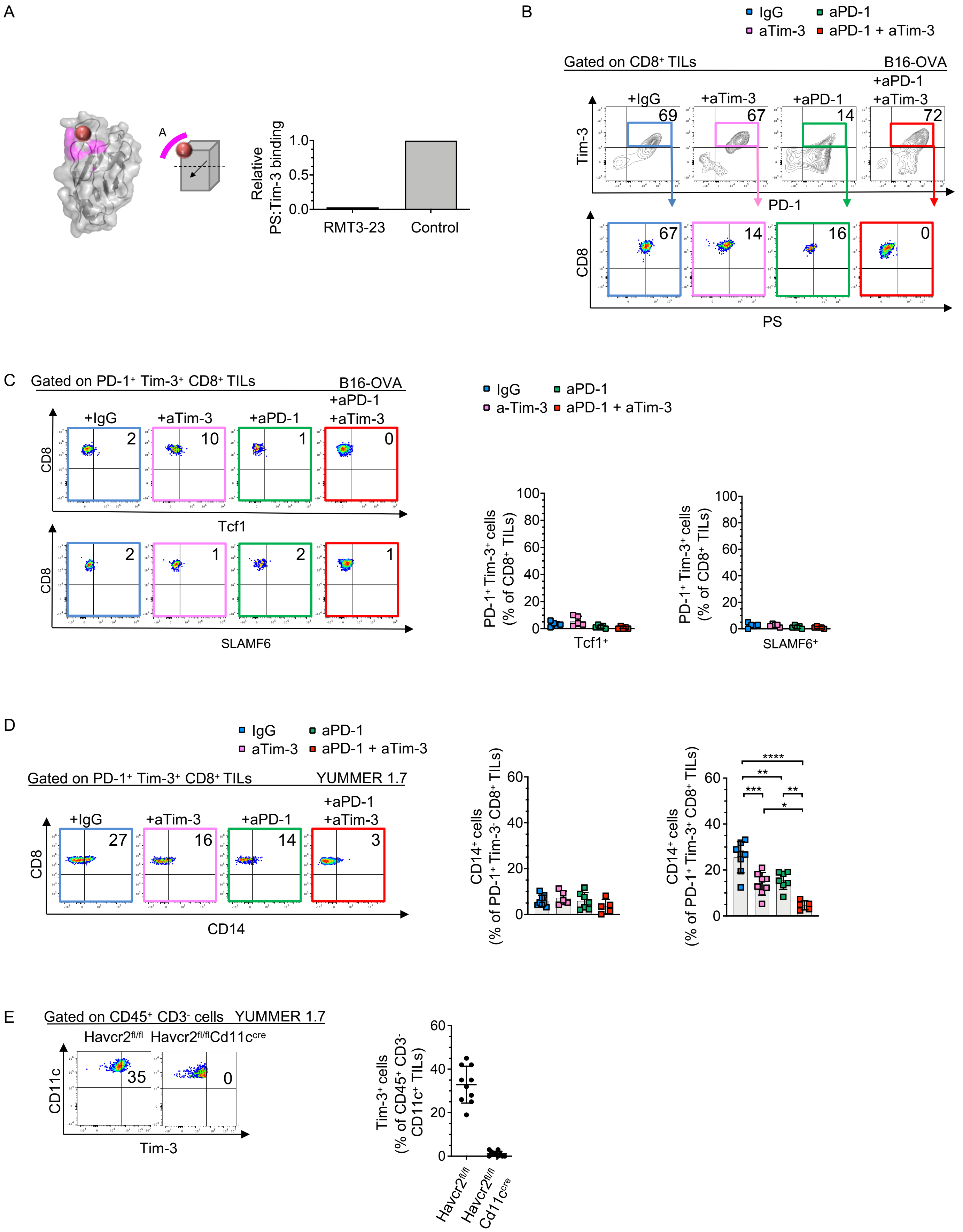

A
Relative PS:Tim-3 binding
RMT3-23
Control
B
IgG
aPD-1
aTim-3
aPD-1 + aTim-3
Gated on CD8+ TILs
B16-OVA
+IgG
+aTim-3
+aPD-1
+aPD-1 +aTim-3
Tim-3
PD-1
CD8
PS
C
Gated on PD-1+ Tim-3+ CD8+ TILs
B16-OVA
Tcf1
SLAMF6
a-Tim-3
PD-1+ Tim-3+ cells (% of CD8+ TILs)
Tcf1+
SLAMF6+
D
Gated on PD-1+ Tim-3+ CD8+ TILs
YUMMER 1.7
CD14
CD14+ cells (% of PD-1+ Tim-3- CD8+ TILs)
CD14+ cells (% of PD-1+ Tim-3+ CD8+ TILs)
E
Gated on CD45+ CD3- cells YUMMER 1.7
Havcr2fl/fl
Havcr2fl/flCd11ccre
CD11c
Tim-3
Tim-3+ cells (% of CD45+ CD3- CD11c+ TILs)

Figure S4

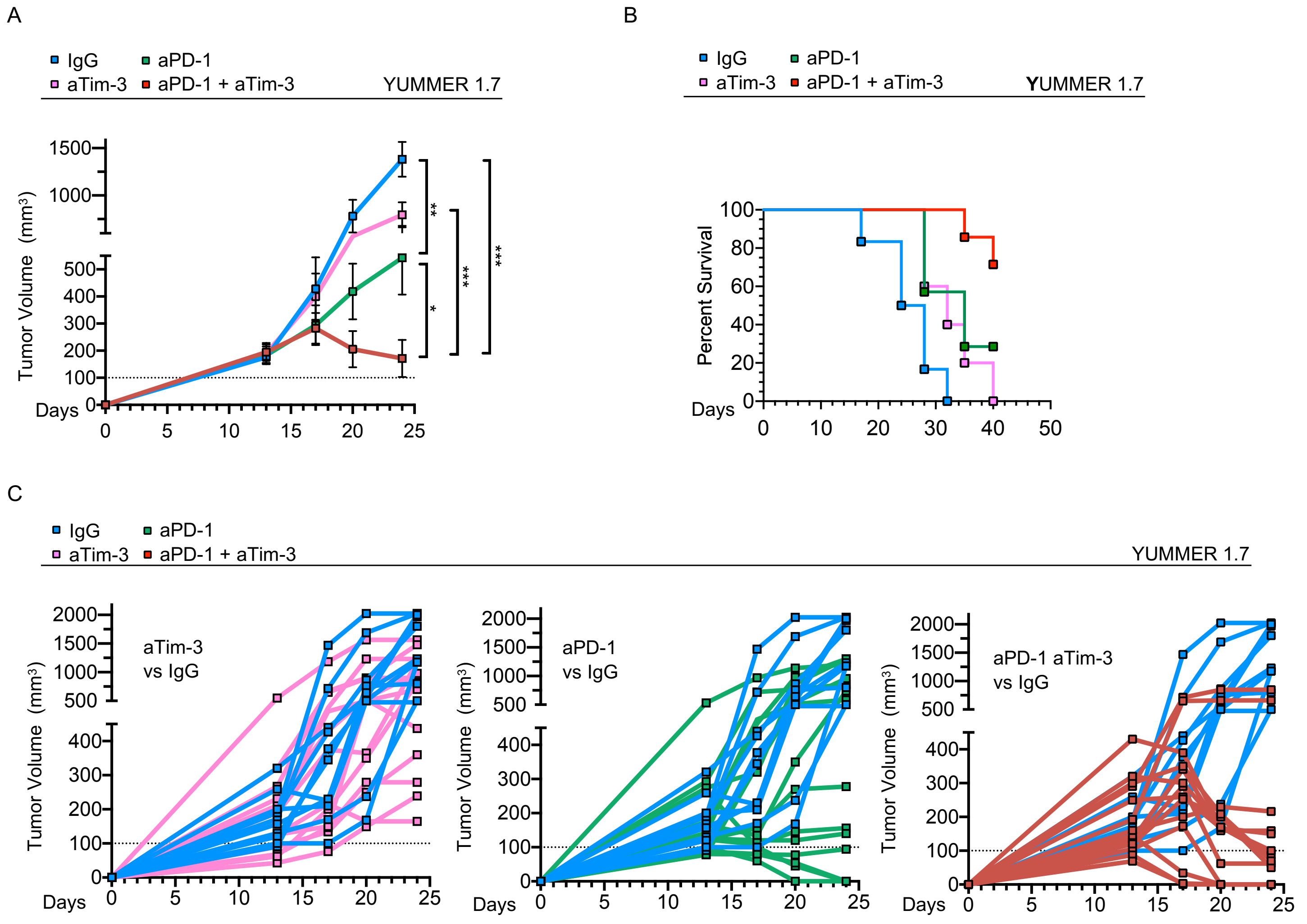
A
IgG
aPD-1
aTim-3
aPD-1 + aTim-3
YUMMER 1.7
Tumor Volume (mm³)
Days
**
*
***
***
B
IgG
aPD-1
aTim-3
aPD-1 + aTim-3
YUMMER 1.7
Percent Survival
Days
C
IgG
aPD-1
aTim-3
aPD-1 + aTim-3
YUMMER 1.7
aTim-3 vs IgG
aPD-1 vs IgG
aPD-1 aTim-3 vs IgG
Tumor Volume (mm³)
Days

Figure S5

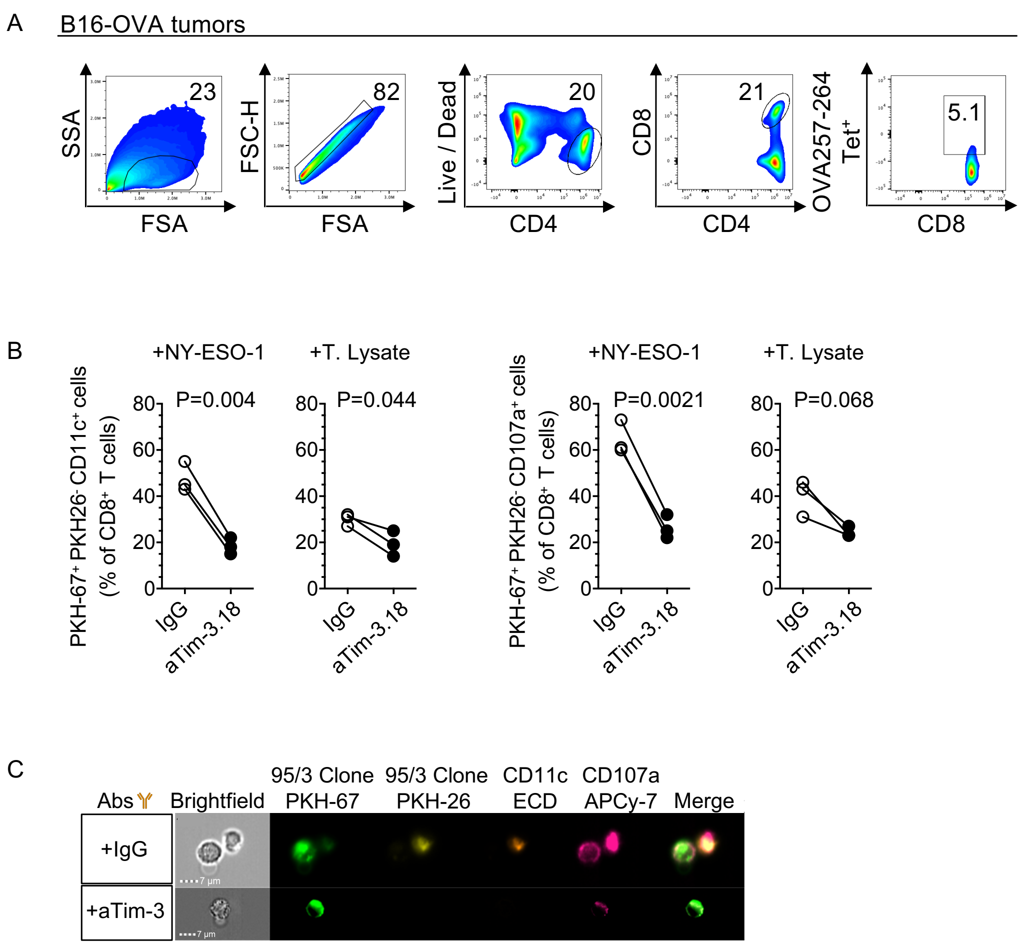
A
B16-OVA tumors
SSA
FSA
23
FSC-H
FSA
82
Live / Dead
CD4
20
CD8
CD4
21
OVA257-264 Tet+
CD8
5.1
B
+NY-ESO-1
+T. Lysate
PKH-67+ PKH26- CD11c+ cells (% of CD8+ T cells)
P=0.004
P=0.044
PKH-67+ PKH26- CD107a+ cells (% of CD8+ T cells)
P=0.0021
P=0.068
80
60
40
20
0
IgG
aTim-3.18
C
Abs
Brightfield
95/3 Clone PKH-67
95/3 Clone PKH-26
CD11c ECD
CD107a APCy-7
Merge
+IgG
+aTim-3

Figure S6

Supplemental Table 1. Top 20 differentially expressed genes identified for the 5 myeloid clusters during the first clustering step.

Myeloid 1			Myeloid 2		
gene	avg_logFC	p_val_adj	gene	avg_logFC	p_val_adj
APOE	3.187285681	0	S100A8	3.699699	0
C1QC	2.9105797	0	LYZ	3.346131	0
SPP1	2.851410092	0	S100A9	3.307772	0
APOC1	2.824481654	0	CST3	2.842015	0
C1QB	2.768863398	0	IFI30	2.58107	0
IL1B	2.586938364	0	FCN1	2.400923	0
TIMP1	2.575994449	0	TIMP1	2.330417	0
C1QA	2.549302238	0	PLAUR	2.217533	0
IFI30	2.547962418	0	TYROBP	2.193984	0
CXCL8	2.516595807	0	FCER1G	2.09211	0
RNASE1	2.464094773	0	IFITM3	2.06748	0
CCL2	2.367808879	0	SERPINA1	2.028154	0
TYROBP	2.340880518	0	AIF1	2.025192	0
CCL3	2.327496789	0	LST1	1.94864	0
CTSB	2.287319137	0	IL1B	1.92112	0
CST3	2.287223474	0	G0S2	1.860119	0
FTL	2.259877145	0	VCAN	1.77685	0
FCER1G	2.221359572	0	CXCL8	1.740869	0
IER3	2.220358691	0	C15orf48	1.704201	0
CTSL	2.182859352	0	CD14	1.691724	0
Myeloid 3			Myeloid 4		
gene	avg_logFC	p_val_adj	gene	avg_logFC	p_val_adj
CST3	1.526552266	0	IL1B	2.994295	0
GPNMB	1.428882141	0	RNASE1	2.903717	0
LYZ	1.160848958	0	CCL3	2.84662	0
CCL2	2.766580853	1.6E-301	APOE	2.721637	0
APOC1	2.099036564	2E-300	APOC1	2.67413	0
RNASE1	1.63982232	1E-290	SPP1	2.668884	0
TGFBI	1.641456982	1.3E-290	CXCL8	2.612032	0
CYP27A1	0.681956822	1.4E-285	C1QB	2.503179	0
APOE	1.84231219	2.7E-284	IER3	2.480689	0
SPP1	2.120742591	8.6E-269	CCL3L1	2.464352	0
CXCL8	1.673091791	5.6E-262	C1QC	2.399699	0
CD68	1.575989253	3.8E-258	C1QA	2.322705	0
ADM	0.979155433	1.5E-254	C3	2.17995	0
CD163	0.943145339	2.5E-243	SGK1	2.1793	0
SMIM25	0.884269662	5.4E-226	TNFAIP2	2.136878	0

TYROBP	1.438477984	5.3E-222	STAB1	2.111032	0
MAFB	1.061970899	8.9E-211	PLAU	1.963226	0
PLPP3	0.416776092	6.4E-210	NUPR1	1.936259	0
FCER1G	1.419444896	4E-209	CXCL2	1.864898	0
CTSL	1.99445162	2.5E-208	TYROBP	1.838547	0
**Myeloid 5**					
**gene**	**avg_logFC**	**p_val_adj**			
APOD	3.554955161	0			
SERPINE2	2.550065669	0			
S100A1	2.49425411	0			
S100B	2.344634122	0			
IFI27	2.211303121	0			
CTSK	2.056528372	0			
S100A13	1.92989759	0			
MIA	1.870078081	0			
PMEL	1.864447283	0			
CRYAB	1.673817698	0			
RAB13	1.530600079	0			
SPARC	1.449488699	0			
MLANA	1.367992397	0			
VGF	1.228123184	0			
CCN3	1.214773447	0			
MITF	1.20955852	0			
BACE2	1.182131031	0			
PLP1	1.177594891	0			
CAPN3	1.169496941	0			
CAV1	1.115816276	0			

Supplemental Table 2. Top 20 differentially expressed genes between the monocyte and cDC clusters.

Monocyte			cDC		
gene	avg_logFC	p_val_adj	gene	avg_logFC	p_val_adj
CTSD	1.130807774	1.45999E-89	CLEC10A	1.03065792	5.6232E-118
FTL	0.685211687	2.83936E-80	PPA1	0.840551508	1.89031E-96
RNASE1	1.85560444	1.11829E-78	CD1C	0.702203859	1.57518E-87
APOE	1.314189697	2.61665E-76	HLA-DRB5	1.160919322	6.25523E-84
SPP1	1.718812346	1.30997E-66	CPVL	0.688056533	4.76053E-79
CCL3	1.241232341	1.21779E-65	PLAC8	0.713944029	1.33584E-75
APOC1	1.392923509	1.21592E-64	FCER1A	0.881101659	1.53815E-73
CXCL8	1.095901872	1.70083E-63	PSME2	0.700576865	1.78576E-71
CCL4	1.373733	5.99083E-60	LIMD2	0.527905119	6.86712E-71
NUPR1	1.547678246	1.1007E-57	PSMB9	0.594811918	4.67969E-67
C1QB	0.833088563	7.71366E-45	HLA-DQA1	0.864073323	1.28825E-66
CTSB	0.626626907	1.57963E-43	CRIP1	0.653559691	2.96479E-66
STAB1	1.126478567	3.00372E-43	RPS23	0.461145762	4.2604E-66
C1QA	0.801926554	1.72589E-41	JAML	0.491021092	7.77405E-65
GPNMB	0.893821952	3.23863E-41	WARS	0.76376329	1.43655E-64
C1QC	0.743782383	4.0114E-41	EEF1A1	0.391400803	1.89465E-64
IL1B	0.81129734	2.39961E-39	HLA-DQB1	0.736702246	2.94173E-62
NEAT1	0.643174976	2.49539E-39	LAP3	0.535145028	6.33087E-61
CCL4L2	1.212316962	6.59087E-39	IFITM1	0.910726568	4.36001E-60
A2M	0.991070547	1.82457E-36	RPL28	0.319764678	5.85742E-60

Supplemental Table 3. Differentially expressed genes between TIM3$^+$ and TIM3$^-$ myeloid cells.

gene	p_val	avg_logFC	pct.1	pct.2	p_val_adj	log10Padj	higher_in
CTSL	3.05783E-27	0.59422444	0.75	0.524	6.71103E-23	22.17321113	TIM3+
HLA-DQB1	4.16535E-25	0.327872767	0.907	0.705	9.14169E-21	20.03897347	TIM3+
TPM4	1.60027E-23	0.269815111	0.703	0.488	3.51212E-19	18.45443047	TIM3+
LITAF	4.62381E-22	0.254061138	0.872	0.662	1.01479E-17	16.99362448	TIM3+
CD63	6.00604E-22	0.257672314	0.858	0.647	1.31814E-17	16.88003695	TIM3+
CTSB	1.07887E-21	0.360495463	0.939	0.819	2.36781E-17	16.62565382	TIM3+
CD163	5.06066E-19	0.27453049	0.403	0.23	1.11066E-14	13.95441759	TIM3+
PLIN2	1.30534E-18	0.27190201	0.535	0.343	2.86484E-14	13.54289976	TIM3+
LGMN	2.4385E-18	0.293714808	0.61	0.41	5.35177E-14	13.2715025	TIM3+
C15orf48	3.29022E-18	0.337644065	0.561	0.372	7.22106E-14	13.14139925	TIM3+
FABP5	2.73296E-16	0.45108647	0.485	0.316	5.99804E-12	11.22199094	TIM3+
IFI30	4.2012E-16	0.284007598	0.981	0.91	9.22038E-12	11.03525126	TIM3+
TIMP1	7.21191E-16	0.411295502	0.699	0.536	1.5828E-11	10.80057475	TIM3+
INSIG1	2.12225E-15	0.292953727	0.577	0.393	4.6577E-11	10.33182856	TIM3+
SDS	1.02503E-14	0.255631941	0.465	0.301	2.24963E-10	9.647889031	TIM3+
CTSD	1.61888E-14	0.310702097	0.858	0.746	3.55296E-10	9.449410087	TIM3+
IFI6	2.20659E-14	0.32531417	0.537	0.368	4.8428E-10	9.314903827	TIM3+
IFITM3	1.92996E-12	0.277972806	0.755	0.571	4.23567E-08	7.37307752	TIM3+
LGALS3	3.27129E-12	0.332920679	0.843	0.694	7.17949E-08	7.14390616	TIM3+
CCL2	3.44491E-12	0.397027542	0.455	0.316	7.56054E-08	7.121447084	TIM3+
CCL7	9.05403E-11	0.485152806	0.101	0.04	1.98709E-06	5.701782921	TIM3+
ISG15	1.57643E-10	0.382714232	0.439	0.304	3.4598E-06	5.460949416	TIM3+
APOBEC3A	2.11962E-10	0.559624249	0.246	0.151	4.65193E-06	5.332367227	TIM3+

Supplemental Table 4. Data collection and refinement statistics.

Wavelength	Tim3.18 Fab-hTim3 complex
Resolution range	48.56 - 1.49 (1.543 -1.49)
Space group	P 1 21 1
Unit cell	66.105 63.856 66.407 90 94.254 90
Total reflections	284783 (29411)
Unique reflections	88986 (8955)
Multiplicity	3.2 (3.3)
Completeness (%)	98.74 (99.62)
Mean I/sigma(I)	13.01 (2.33)
Wilson B-factor	18.75
R-merge	0.05611 (0.4747)
R-meas	0.06776 (0.5676)
R-pim	0.03752 (0.3076)
CC1/2	0.99 (0.884)
CC*	0.997 (0.969)
Reflections used in refinement	88901 (8932)
Reflections used for R-free	4367 (463)
R-work	0.1770 (0.2564)
R-free	0.2013 (0.2955)
CC(work)	0.963 (0.908)
CC(free)	0.965 (0.844)
Number of non-hydrogen atoms	4890
macromolecules	4222
ligands	24
solvent	644
Protein residues	544
RMS(bonds)	0.009
RMS(angles)	1.34
Ramachandran favored (%)	97.39
Ramachandran allowed (%)	2.61
Ramachandran outliers (%)	0
Rotamer outliers (%)	0
Clashscore	5.01

**Average B-factor**	29.73
**macromolecules**	28.21
**ligands**	40.71
**solvent**	39.33
**Number of TLS groups**	20

Statistics for the highest-resolution shell are shown in parentheses.

Supplemental Table 5. Determination of aTim-3 antibody binding kinetics and affinities via surface plasmon resonance.

Antibody	Analyte	ka (1/Ms)	kd (1/s)	KD (M)
RMT3-23	mTim3	9.80E+05	2.70E-02	2.80E-08
Tim 3.18	hTim3	2.60E+06	4.60E-03	1.80E-09
Tim 3.22	hTim3	2.80E+06	1.40E-03	5.10E-10
